# Structure of the mini-RNA-guided endonuclease CRISPR-Cas12j3

**DOI:** 10.1038/s41467-021-24707-3

**Published:** 2021-07-22

**Authors:** Arturo Carabias, Anders Fuglsang, Piero Temperini, Tillmann Pape, Nicholas Sofos, Stefano Stella, Simon Erlendsson, Guillermo Montoya

**Affiliations:** 1 Structural Molecular Biology Group, Novo Nordisk Foundation Centre for Protein Research, Faculty of Health and Medical Sciences University of Copenhagen, Copenhagen, Denmark; 2 Core Facility for Integrated Microscopy (CFIM), Faculty of Health and Medical Sciences, University of Copenhagen, Copenhagen, Denmark; 3 Structural Studies Division, MRC Laboratory of Molecular Biology, Cambridge, UK; 4 Present Address: Twelve Bio ApS, Copenhagen, Denmark

**Keywords:** Structural biology, CRISPR-Cas systems, Cryoelectron microscopy

## Abstract

CRISPR-Cas12j is a recently identified family of miniaturized RNA-guided endonucleases from phages. These ribonucleoproteins provide a compact scaffold gathering all key activities of a genome editing tool. We provide the first structural insight into the Cas12j family by determining the cryoEM structure of Cas12j3/R-loop complex after DNA cleavage. The structure reveals the machinery for PAM recognition, hybrid assembly and DNA cleavage. The crRNA-DNA hybrid is directed to the stop domain that splits the hybrid, guiding the T-strand towards the catalytic site. The conserved RuvC insertion is anchored in the stop domain and interacts along the phosphate backbone of the crRNA in the hybrid. The assembly of a hybrid longer than 12-nt activates catalysis through key functional residues in the RuvC insertion. Our findings suggest why Cas12j unleashes unspecific ssDNA degradation after activation. A site-directed mutagenesis analysis supports the DNA cutting mechanism, providing new avenues to redesign CRISPR-Cas12j nucleases for genome editing.

## Abstract (web summary)

The Class 2 family of CRISPR nucleases named Cas12j, which shares only low sequence identity with other CRISPR nucleases was recently identified in the biggiephage clade of phages. Here, the authors present the cryo-EM structure of a functional Cas12j3−crRNA complex in the post-catalytic state and discuss Cas12j3 PAM recognition, hybrid stabilisation and the activation mechanism.

## Introduction

Competition between microbes and their invaders has driven the evolution of defence systems preventing the attack of mobile genetic elements. Among them, CRISPR constitutes a type of adaptive immunity achieved by CRISPR-associated nucleases (Cas) and CRISPR RNAs (crRNAs) that assemble effector RNPs, which are guided by the crRNA to cleave complementary DNA (or RNA) for interference^1–3^. Depending on the type of RNP effector nucleases, CRISPR-Cas systems can be categorized into two classes (1 and 2), which are further subdivided into six types (types I through VI). Class 2 systems form an RNP complex between the multidomain effector Cas protein and a CRISPR RNA (crRNA) that contains the necessary information to target a specific nucleic acid sequence^2,4,5^.

Although ubiquitously diversified among prokaryotes, CRISPR systems were also identified in bacteriophages^6^. Recently, a new Class 2 family of CRISPR nucleases named Cas12j, also known as CasΦ, was found in the biggiephage clade of phages^7^. Cas12j proteins share low sequence identity with other CRISPR nucleases and display sequence homology only in their RuvC domain with Class2 type V members. They generate a staggered DNA double-strand break (DSB) and unleash unspecific ssDNA cleavage after activation with an ssDNA complementary to the crRNA, as other members of Class 2 type V^7,8^. In addition, the RuvC catalytic site of Cas12j1 and 2 also processes the precursor crRNA (pre-crRNA)^7^. This family of endonucleases recognise protospacers with a minimal T-rich PAM, and their small size (700–800 residues) together with the lack of a transactivation crRNA (trac-crRNA) to build the functional RNP, make Cas12j an atypical family of miniaturized RNA-guided nucleases (Supplementary Fig. 1).

Cas9 is the best-characterized member of Class 2 (Type II-A) and it has been developed into a genome editing tool^9–15^. Diverse Cas9 nuclease variants are involved in clinical trials to develop new therapies against human diseases^16^. The redesign of the CRISPR-Cas guide RNA, as well as the modification of the protein^17,18^, has provided a powerful method for genome editing in biomedical and biotechnological applications^19,20^. In some of these applications Adeno-associated viral (AAV) vectors are commonly used for gene delivery. Yet, packaging of the genes coding for CRISPR-Cas effector complexes into an AAV vector is challenging due to its limited capacity, thus leaving little space for the insertion of additional regulatory elements. Recently, Cas12j enzymes have been shown to mediate genome editing in mammalian and plant cells^7^ expanding our *repertoire* of genome manipulation tools. The small size Cas12j RNPs can improve our genome editing approaches by alleviating the packing problems in the AAV vectors used for delivery^20^. However, questions regarding the detailed molecular mechanism of target DNA cleavage by Cas12j nucleases remain unanswered, as no structural information is available.

Here we report the structure of Cas12j3 in complex with a target DNA after cleavage, revealing the key regions involved in the PAM recognition, hybrid stabilization and activation of the catalysis. Our work sheds light on the mechanism of action of the Cas12j family, facilitating the redesign of CRISPR-Cas12j nucleases for genome editing.

## Results

### Cas12j3 characterisation

**Fig. 1:**
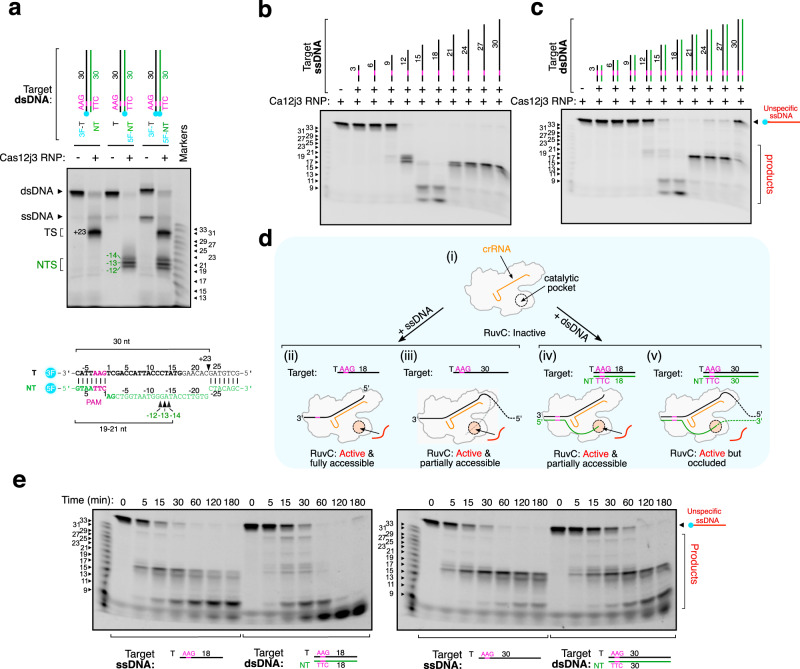
Cas12j3 target dsDNA cleavage and unspecific ssDNA degradation. **a** representative dsDNA cleavage pattern generated by Cas12j3 wild type (WT). T-strand (TS) and NT-strand (NTS) products are marked, showing a cut at positions −13, −14 and −15 of the NT-strand, while the T-strand is cleaved at position +23. The sequence of the double-labelled duplex is shown below, marking the position of the cut (triangles), and the size of the labelled products. **b** Unspecific ssDNA degradation after activation with a specific target ssDNA of different lengths (Oligonucleotides T-AAG-3 to T-AAG-30 in Supplementary Table II). **c** Unspecific ssDNA degradation after activation with a specific dsDNA activator of different lengths (Oligonucleotides T-AAG-3/NT-TTC-3 to T-AAG-30/NT-TTC-30 in Supplementary Table II). **d** Schematic cartoon of the results shown in b) and c). Activation of the unspecific ssDNA cleavage is observed between 12–30 nt. (i) The RuvC domain of Cas12j3 RNP is inhibited. Full activation of the unspecific cleavage is observed when using an ssDNA or dsDNA activator pairing with the crRNA between 12–18 nt (ii and iv). The use of longer oligos as ssDNA(iii) or dsDNA (v) results in a reduction of the cleavage efficiency, likely due to a steric occlusion of the catalytic site by the T-strand and NT-strand. **e** Unspecific ssDNA degradation time course by Cas12j3 activated by 18 and 30 nt, ss- and dsDNA targets. The DNA markers indicate the size of the products in nucleotides. The experiments are shown in **a**–**c** and **e** are representative of three independent experiments with similar results.

**Fig. 2:**
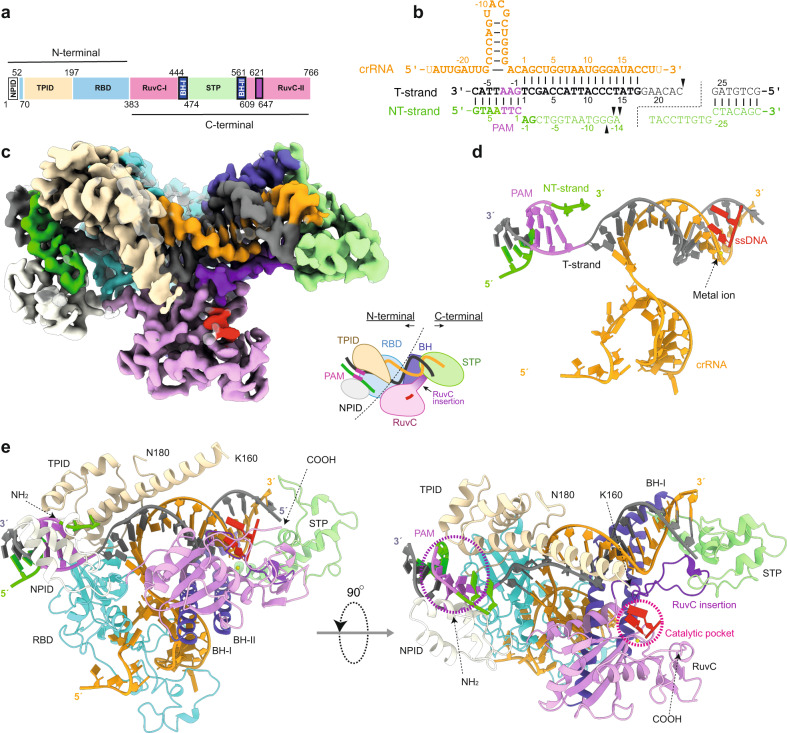
Cryo-EM structure of Cas12j3 R-loop complex after target DNA cleavage. **a** Domain architecture of Cas12j3 comprising the T-strand and NT-strand PAM interacting domains (TPID, NPID), the RNA-handle binding domain (RBD), the bridge helices (BH-I and BH-II), the RuvC domain including the insertion and the stop (STP) domain. **b** Schematic diagram of the R-loop formed by the crRNA and the target DNA. Triangles represent phosphodiester cleavage positions in the T- and NT-strands; the bold font nucleotides represent those visualized in the structure. The PAM distal products are separated by a dashed line to illustrate the post-catalytic state (see Supplementary Fig. 7c–g). **c** cryo-EM map of the Cas12j3/R-loop complex at 2.7 Å resolution. The cartoon depicts the relative orientation of the domains and the N- and C-terminal regions of the protein. The map and the explicative cartoon are coloured according to Fig. 2a. **d** View of the R-loop structure and the dinucleotide in the catalytic site (polypeptide omitted). **e** Overview of the Cas12j3–RNA–target-DNA ternary complex (Supplementary Figs. 3–5, Supplementary Table 1).

**Fig. 3:**
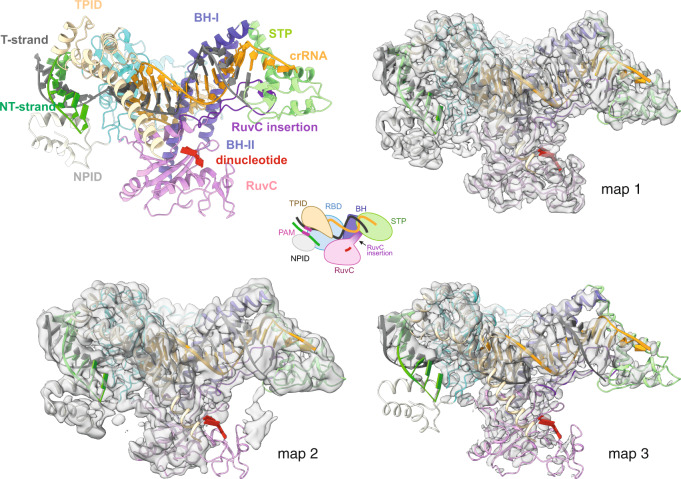
Cas12j3/R-loop atomic model and cryo-EM maps. Comparison of the three cryo-EM maps (Supplementary Fig. 3d, Supplementary Fig. 4 and Supplementary Table 1). The top left ribbon diagram and the central cartoon are included to facilitate the comparison between the maps. The maps indicate the high flexibility of the NPID, RuvC and STP domains and extra density from the T-strand in map2 (Supplementary Movie 1).

We reconstituted a functional Cas12j3−crRNA complex and determined the structure of the enzyme after severing a target dsDNA by cryo-EM (Figs. 1–2, Supplementary Figs. 2–4, Supplementary Table 1, Methods). Cas12j3 generates an overhang of 9-11-nt by cleaving at different phosphodiester bonds (Fig. 1a, Fig. 2b). A collateral effect of target DNA cleavage is the release of indiscriminate ssDNA degradation^7^, which is also triggered by a ssDNA complementary to the crRNA mimicking the T-strand (Fig. 1b–c) similarly to Cas12a^21^. In both cases, indiscriminate cleavage is unleashed when a minimal 12-15-nt crRNA-DNA duplex is assembled. One of the cryoEM maps (map 2) suggests that the differences observed with DNA activators longer than 18-nt can be attributed to the presence of the R-loop disturbing the entrance of the unspecific ssDNA substrate in the catalytic site (Figs. 1d, 3 map2 and Supplementary Movie 1). The appearance of different cleavage products with activators of different lengths might suggest a change in the cleavage pattern. To address this question, we performed a time-course experiment using representative target DNAs (Fig. 2e). The assay suggests that the different lengths observed in the cleavage products are related to the different cutting efficiencies, as the same bands are observed at different time points for all the activators. The activity was tested in the presence of Mg^2+^ and other divalent cations (Supplementary Fig. 2a). The assay revealed that Mg^2+^, Mn^2+^, Fe^2+^, Co^2+^, and Ni^2+^ support Cas12j3 catalysis, generating different cleavage products. Cas12j3 activity was saturated when the endonuclease/DNA ratio was nearly equimolar, suggesting the slow dissociation of the enzyme from the PAM-proximal cleavage product, as observed in other RNA-guided nucleases^22,23^ (Supplementary Fig. 2b). In addition, removing the final 39 residues, not visualized in the structure, decreased Cas12j3 activity (Supplementary Fig. 2c–d).

### Structure of the Cas12j3/R-loop complex

Heterogeneous refinement resulted in the visualisation of several conformations of the complex. The predominant class yielded a map at a resolution of 2.7 Å, which was used to build the model of the Cas12j3/R-loop structure. The high flexibility observed in other predominant classes precluded model building but revealed the flexible regions and the conformational heterogeneity of the complex, which we further supported by performing a 3D variability analysis^24^ (Figs. 2–3, Supplementary Figs. 3-4, Supplementary Table 1, Supplementary Movie 1, Methods).

The Cas12j3/R-loop complex does not present the classical bilobal architecture observed in other type V effector complexes^25^. The R-loop displays a T shape with the crRNA/DNA hybrid and the crRNA handle forming the horizontal and vertical bars, and the protein domains wrapping around the nucleic acids (Fig. 2d–e). The handle of the crRNA is stabilized by the strictly conserved R338 which interacts with C-1 and U-18 and the neighbouring non-canonical Watson-Crick base pair interaction between G-17 and A-2 (Supplementary Fig. 6). The PAM- proximal and  distal regions of the heteroduplex are recognized by the N- and C-terminal regions of the protein (Fig. 2a, d, e), which are connected by a 15-residue loop (380–395). Each region comprises around half of the size of the protein and they are separated by the long handle of the crRNA on the T-shape assembly. The N-terminal region comprises the T-strand and NT-strand PAM interacting domains (TPID, NPID) and the RNA-handle binding domain (RBD), while the C-terminal consists of the catalytic RuvC and stop (STP) domains (Fig. 2a). The RuvC domain is split into RuvC-I and RuvC-II by the presence of the STP domain, which is connected to the catalytic domain by two long bridge helices, BH-I and BH-II. Additionally, the RuvC-II subdomain includes a characteristic insertion, which is conserved in all Cas12j except Cas12j6 (Fig. 2a, Supplementary Fig. 1). The N- and C-terminal separation seems to be also functional, as the domains involved in RNP assembly, PAM recognition and unwinding reside in the N-terminal section, while those stabilizing the crRNA/T-strand hybrid and catalysis of the target DNA are located in the C-terminal region of the polypeptide. Therefore, the PAM site is ~ 55 Å away from the RuvC nuclease active site.

**Fig. 4:**
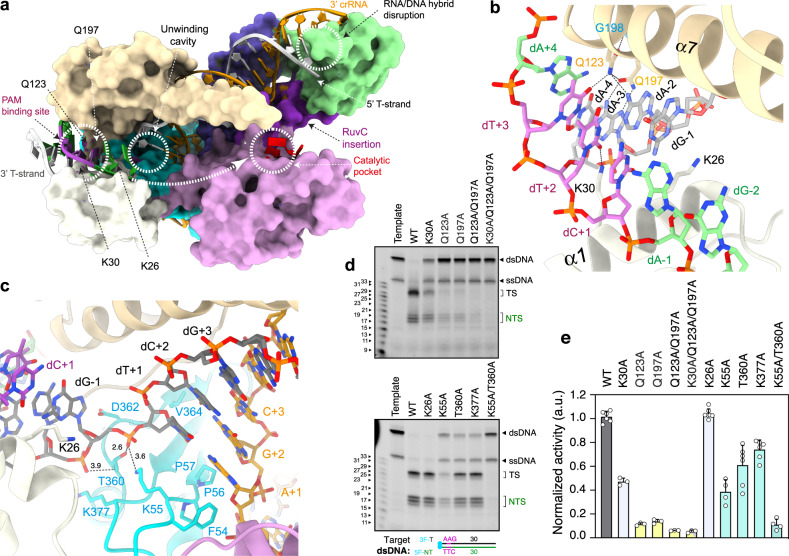
Cas12j3 PAM recognition, uncoupling of the Watson–Crick dA-1:dT+1 pair and unzipping. **a** Surface representation of Cass12j3–R-loop complex. The white dashed arrow shows the predicted path of the NT-strand to the nuclease site after dG-2 (Supplementary Fig. 6). **b** Detailed view of the PAM nucleotides recognition and dsDNA unwinding depicting the conserved K26, K30, Q123, and Q197 residues (Supplementary Fig. 5c). **c** Zoom of the dT+1/dA-1 pair uncoupling, phosphate inversion and unzipping (Supplementary Fig. 5d). **d** dsDNA cleavage assays using Cas12j3 wild type and PAM unwinding, activation and catalytic mutants. Oligonucleotides 3F-T-AAG-30 and 5F-NT-TTC-30 were used as substrates (Supplementary Table II). T-strand (TS) and NT-strand (NTS) products are marked. DNA markers are shown in nucleotides. **e** Quantification of the activity based on the cleavage experiments as shown in **d**. Bars represent mean ± SD. *n* > 3 independent experiments.

The target DNA cleavage yields a triple strand R-loop with the T-strand hybridized to the crRNA (Fig. 2b, d), while the dissociated PAM NT-strand is directed towards the catalytic pocket (Fig. 4a). The NT-strand nucleotides −1 to −2 upstream of the PAM were built in the density but the high flexibility on its distal end precluded visualization of the rest, as shown for Cas9^26^ and Cas12a^22^. Nevertheless, the backbone of the NT-strand is observed at a low contour level in the cryo-EM maps, suggesting the path followed by the DNA to the RuvC catalytic pocket (Supplementary Fig. 7a–b, Supplementary Movie 1). Interestingly, two nucleotides, modelled as purines, were observed in the active site in complex with Ni^2+^ as a by-product of the phosphodiester hydrolysis (Fig. 2c–e, Supplementary Fig. 6, Methods). To determine to which strand these nucleotides belong, we performed a binding assay after cleavage with different labelled target DNAs, revealing that these nucleotides originate from the NT-strand (Supplementary Fig. 7c–g).

### PAM recognition

PAM recognition is an important aspect of DNA targeting by CRISPR-Cas nucleases, as it is a prerequisite for target DNA identification, strand separation and crRNA–target-DNA heteroduplex formation^27^ before cleavage. Cas12j3 recognizes a 5′-TTN-3′ PAM sequence in the NT-strand^7^. Our structure shows that PAM recognition in Cas12j3 is achieved by combining interactions in both strands by the TPID and NPID domains (Fig. 4b, Supplementary Fig. 5b, Supplementary Fig. 6). The positively charged side of helix α1 (S21 to A34) in the NPID is inserted in the minor groove at an angle of 45° with respect to the dsDNA longitudinal axis. The conserved K26 and K30, interact with the NT-strand. K30 makes contact with dT+2, while K26 is placed inside the dsDNA disrupting the Watson-Crick base coupling, displacing the NT-strand and stabilizing the separation (Fig. 4b–c, Supplementary Fig. 5b–c). On the other side of the PAM recognition cleft, Q123 in the TPID builds an intricate network of polar interactions with dA-3 and dA-2 in the T-strand and the dT+3 in the NT-strand (Fig. 4b, Supplementary Fig. 6). The neighbouring G198 amide contacts the carbonyl of Q123, anchoring the side chain in a conformation favouring the contacts with these bases. In addition, the side chain of Q197 interacts with Q123 and hydrogen bonds with dA-3.

The Q123A and Q197A mutations present ~90% activity reduction, while the K30A mutant reduces cleavage by ~55%. The triple mutant activity is similar to the Q123A/Q197A mutant, indicating the pivotal role of the glutamines in PAM recognition, as the addition of the K30A mutation does not display a further reduction (Fig. 4d–e). The K26A mutant does not affect activity, suggesting that unzipping may proceed without the insertion of this residue and that the observed conformation of the helix α1 stabilizes the unzipped dsDNA after unwinding in the post-catalytic state. None of these mutants changed the cleavage pattern (Fig. 4d–e). However, neither wild type nor the mutants cleaved target dsDNAs with different PAM sequences or in the absence of PAM, underscoring the selectivity of the PAM interaction network formed by Q123A, Q197A, and K30A (Supplementary Fig. 8a).

Next, we assessed the role of the PAM complementary bases in the T-strand by triggering the unspecific activity of Cas12j3 using ssDNAs activators mimicking the T-strand with different PAM sequences (Supplementary Fig. 8b). As expected, the unspecific ssDNA catalysis is also activated in the presence of target dsDNA, suggesting that after PAM recognition crRNA/DNA hybrid assembly activates catalysis (Fig. 1c, Supplementary Fig. 8b) in a fashion similar to Cas12a^21^. To assess the role of the PAM complementary bases in the T-strand, we triggered the unspecific activity of Cas12j3 using ssDNA activators with different PAM complementary sequences. The assay showed that the PAM complementary 3′-AAG-5′ and an activator without PAM unleashed phosphodiester hydrolysis, while other sequences promoted no or lower levels of activation, suggesting that the assembly of a hybrid duplex longer than 12-nt unleashes catalysis (Fig. 1b–c, Supplementary Fig. 8b). Activators containing regions that build shorter duplexes or partially hybridize with the crRNA display lower cleavage (Supplementary Fig. 8b).

Collectively, the combined structure-function analysis suggests that the well-conserved Q123 and Q197 residues play an essential role in PAM recognition. The direct base readout in the PAM region of Cas12j nucleases combine interactions of the TPID and NPID with both strands of the target DNA, and the interactions of the TPID with the T-strand seem to have an important role in PAM discrimination.

### The unwound T-strand is stabilized by hybrid assembly

Overlaying with the PAM site, the antiparallel β-sheet consisting of the RBD β1, β6 and β7 (Supplementary Fig. 1), builds a cavity where the dissociated T-strand is stabilized by the hybridisation with the crRNA in the post-catalytic stage (Fig. 4c). This cavity is flanked on the C-terminal region by the BH-I helix and the RuvC domain. The well-conserved F54, K55, P56, P57, P363, T360, G361, D362, and V364 organize the cavity combining acidic and hydrophobic residues facilitating the Watson-Crick coupling of dT+1 and A + 1 between the T-strand and the crRNA (Fig. 4c, Supplementary Fig. 5c, Supplementary Fig. 6). In addition, the backbone phosphate group of dG-1 is recognized by the side chain of the T360, K55 and the main chain of Y376. This interaction results in the rotation of the phosphate group (Fig. 4c, Supplementary Fig. 6), thus facilitating base pairing between dT+1 and A + 1 in the crRNA as observed in other CRISPR-Cas nucleases^21,22,28–31^. The neighbouring K377A mutation led to a ~20% decrease, while the T360A and K55A mutations reduced the activity by 50 and 60%. However, the double mutant activity is ~10%, highlighting the importance of these residues for hybrid formation (Fig. 4d–e).

In the post-catalytic cleavage state represented in our structure, helix α7 in the TPID directs the crRNA/T-strand hybrid into the nest formed by the BH-I and II and the RuvC insertion, detaching the hybrid from the NT-strand and preventing a possible reannealing. The conformation of α7 observed in our structure complicates the initial assembly of the hybrid, as the T-strand would need to access through a narrow passage in order to anneal with the crRNA. The region of the TPID comprising α7 seems rather flexible, as we cannot visualize 20 residues (160–180), which connect α7 to the rest of the TPID (Fig. 2e). This observation is also supported by the 3D variability analysis of our structure (Supplementary Movie 1). Therefore, we suggest that the conformation of α7 in the apo and pre-catalytic stages should be different to the configuration observed in our post-catalytic state structure, in order to facilitate the initial stages of unwinding and hybrid formation.

**Fig. 5:**
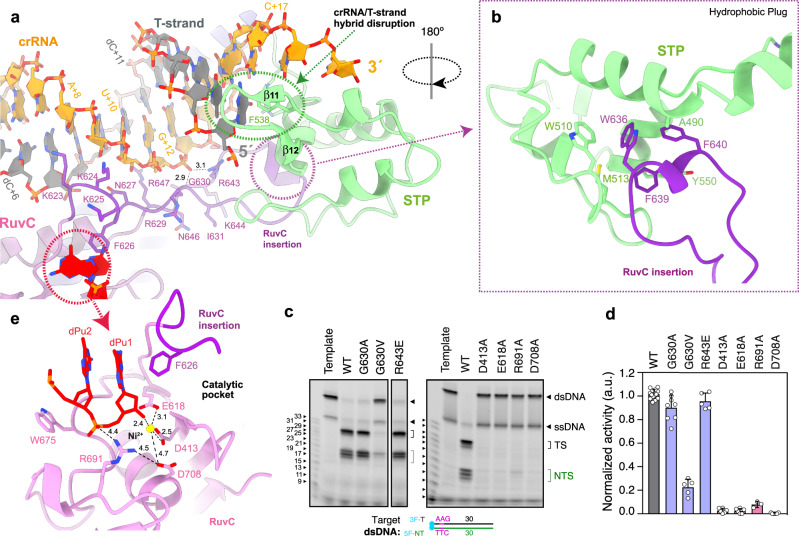
Assembly of the crRNA/DNA hybrid and activation of the RuvC pocket. **a** View of the hybrid showing the interaction of the crRNA with residues in the RuvC insertion (Supplementary Fig. 5d). **b** Inset depicting the hydrophobic interaction between the turn of the RuvC insertion and the and cavity in the STP domain (Supplementary Fig. 5f). **c** dsDNA cleavage assays using Cas12j3 wild type and PAM unwinding, activation and catalytic mutants. Oligonucleotides 3F-T-AAG-30 and 5F-NT-TTC-30 were used as substrates (Supplementary Table II). T-strand (TS) and NT-strand (NTS) products are marked. DNA markers are shown in nucleotides. **d** Quantification of the activity based on the cleavage experiments as shown in **c** Bars represent the mean ± SD. *n* > 3 independent experiments. **e** Detailed view of the catalytic site containing a dinucleotide and a divalent metal. The D708 side chain and the associated distances are shown for visualization purposes only (see Supplementary Fig. 5f).

The area where the hybrid rests is flanked by the catalytic RuvC and STP domains, which disrupts the crRNA/T-strand hybrid as a vessel bulb bow (Fig. 5a). An antiparallel β-sheet formed by β11 and β12 splits the Watson-Crick base coupling after the dG+17:C + 17 pair; thus, limiting the hybrid length to 17 nucleotides in agreement with cleavage experiments testing the efficiency of the spacer length^7^. The aromatic ring of F538 in β11 promotes hybrid unzipping (Fig. 5a), This chemical trick to disrupt the duplex is also observed in Cas12a^22,30,31^. However, the arrangement of the protein moiety in Cas12a and Cas12j3 is different. While in Cas12a the REC2 domain acts like the stop at the end of the train track, in Cas12j3 the STP domain splits the duplex as a blade separating the crRNA and the T-strand. The 3′-phosphate of the crRNA is guided to the back side of the domain where C + 17 and U + 18 are accommodated by a combination of basic (R535, R547) and hydrophobic residues (M500, L555). The 5′-phosphate of the T-strand is directed to the opposite side where the RuvC catalytic pocket is located.

### Activation of catalysis

The RuvC insertion runs alongside the crRNA strand of the hybrid, making multiple contacts with its phosphate backbone from U + 9 to G + 13 (Fig. 5a, Supplementary Fig. 6, Supplementary Fig. 5d). The turn at the tip of the RuvC insertion, comprising the conserved W636, F639 and F640 hydrophobic residues, is anchored in a cleft of the STP domain, composed also by the conserved hydrophobic A490, W510, M513 residues (Fig. 5b, Supplementary Fig. 5e). This arrangement together with the activity assays (Fig. 1b–c), suggest that hybrid assembly could trigger changes in the RuvC domain transmitted through the insertion, thus activating catalysis by making the catalytic pocket available for the substrate. The monitoring of the unspecific ssDNA cleavage using activators of different lengths (Fig. 1b–c), shows that this activity of Cas12j3 is fully released when the activator builds a 12-nt crRNA/DNA hybrid or longer, supporting the notion that a certain duplex length stimulates catalysis. The conserved G630 and R643 are key residues organizing a network of polar interactions with the G + 12 phosphate (Supplementary Fig. 1), resulting in a special organisation of connections observed in our post-catalytic state structure (Fig. 5a). We hypothesize that the assembly of the hybrid and its interaction with the RuvC insertion could trigger the activity. Additionally, the conformations observed in the 3D variability analysis suggests positioning the STP domain towards the catalytic site facilitating the T-strand access to the active site with the proper 5′–3′polarity (Supplementary Movie 1).

We analysed substitutions in G630 and R643 that could destabilize the interaction of the hybrid with the RuvC insertion. The G630A mutation exhibited a minor activity decrease ~10% (Fig. 5c–d), in agreement with the G630 contribution to the polar network through its main chain. However, the G630V substitution displayed a strong reduction, suggesting that a bulkier side chain would destabilize the interaction with the G + 12 phosphate. Interestingly, the reversed polarity mutant R643E presented a minimal cleavage reduction of the target dsDNA (Fig. 5c–d), however, its indiscriminate ssDNA degradation activity showed ~100% reduction, as did the G630V mutant (Supplementary Fig. 8c–d). Therefore, substitutions destabilizing the RuvC insertion can abrogate DNA cleavage and also modify the severing properties of Cas12j3, suggesting that mutations in this conserved region could be used to redesign the Cas12j family of endonucleases.

In addition, all the PAM and phosphate inversion mutants, where the target DNA is not recognized and the hybrid cannot be stabilized, display indiscriminate ssDNA activity when the same assay was performed using an ssDNA activator lacking the PAM (Supplementary Fig. 8c–d). This activator would skip recognition and unwinding, thus hybridising with the complementary crRNA and triggering activation. However, when the PAM is present in the target dsDNA the variants displayed a minimal activity, as their PAM recognition and hybrid assembly are compromised, in agreement with their specific target dsDNA cleavage activity (Fig. 4d–e). These results support the proposed model, as the PAM and phosphate inversion mutants would skip recognition and unwinding when activated with ssDNA, thus hybridising with the crRNA and triggering the nuclease activity.

Collectively, the data suggest that PAM recognition, DNA unwinding and activation are linked in the presence of a target dsDNA, while catalytic activation can omit PAM recognition if a suitable ssDNA is provided. Furthermore, mutations in the section of the RuvC insertion interacting with the hybrid affect the enzyme activity and change its pattern as observed in the case of the G630V and R643E mutants. Finally, substitutions in this region can also dissociate the indiscriminate ssDNA activity from the specific target dsDNA cleavage (Fig. 5c–d, Supplementary Fig. 8c–d).

### Cas12j3 catalytic site

**Fig. 6:**
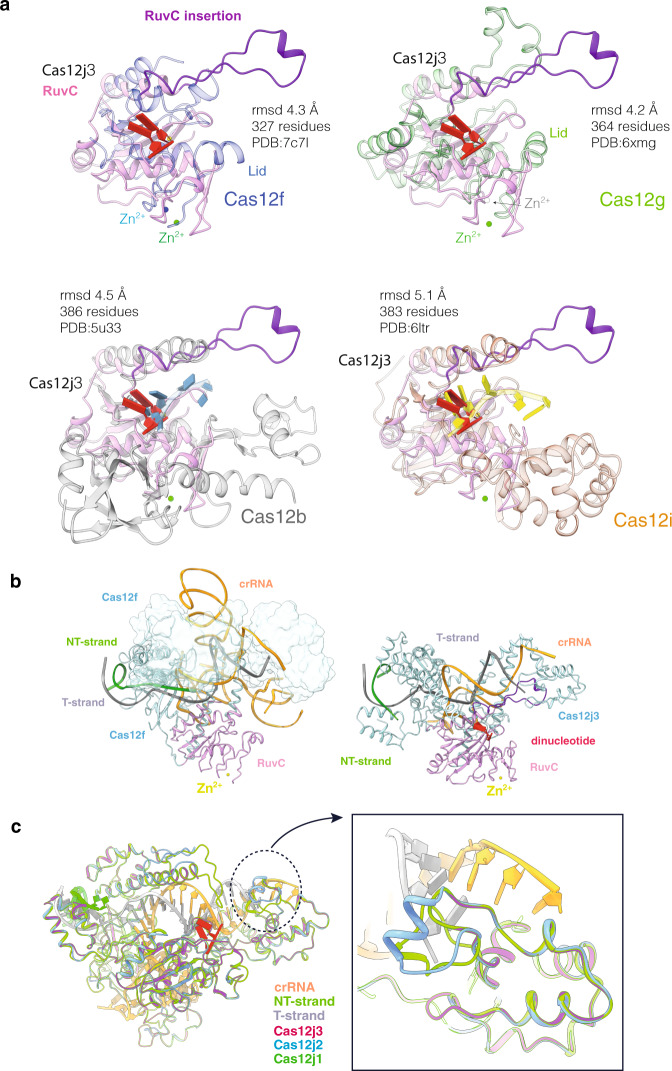
Structural comparison of Cas12j3 RuvC domain with other Cas nucleases. A structural homology search of Cas12j3 against the PDB was performed using DALI^39^. Only the RuvC domain displays homology with other Cas nucleases. **a** The top panel shows the superposition of Cas12j3 with Cas12f and Cas12g. Both Cas12f and Cas12g present a Zn^2+^ atom coordinated by 4 conserved cysteines as Cas12j3. The rest of the domain is different to the TNB domain in Cas12f and Cas12g. Bottom panel Superposition of Cas12j3 with and Cas12i. Both Cas12b and Cas12i present DNA in the catalytic site and the Nuc domain inserted in the RuvC. **b** Detailed comparison of Cas12j3 and Cas12f after superposition in the RuvC domain. One of the monomers of the dimeric Cas12f is shown in surface representation for clarity. **c** Homology modelling of Cas12j1 and Cas12j using Cas12j3 and superposition of the three Cas12j family members. The inset shows the differences in the STP domain.

The RuvC domain of Cas12j nucleases belongs to the retroviral integrase superfamily that displays a characteristic RNaseH fold. The two nucleotides from the NT-strand in the catalytic Cas12j3 pocket are associated with the conserved E618 and D413 (Fig. 5e). The density did not allow base identification, and either dA or dG could be modelled. We built two guanines with a 5′–3′ polarity and a Ni^2+^ ion in the density, in agreement with the number of nucleotides in the cleavage products and the purine-rich sequence in that position (Methods, Fig. 2b, Fig. 5e, Supplementary Figs. 6, 7). The length of the DNA after DSB generation could permit that the cleaved NT-strand remains associated with the catalytic centre and may disturb the entrance of the T-strand delaying its catalysis^7^ (Fig. 1e, Supplementary Fig. 7, Supplementary Movie 1). A second metal atom, modelled as Zn, is coordinated by 4 conserved cysteines, similarly to Cas12g^32^ and the dimeric Cas12f^33^ (Fig. 6a–b). This section of RuvC includes the conserved R691 4.4 Å away from the dinucleotide, whose mutation abrogates activity (Fig. 5c–d). This residue could facilitate the positioning of the phosphodiester backbone in the catalytic pocket for catalysis (Fig. 5e). However, the rest of this region is different to the target nucleic acid-binding (TNB) domain in Cas12f and Cas12g (also known as Nuc for Cas12a and Cas12b and the target-strand loading domain for Cas12e), as it displays a different structure without the helical regulatory lid motif (Fig. 6a).

RuvC domains introduce 5′-phosphorylated cuts and involve three acidic amino acids^34^ and two divalent metal ions^35^. The E618 and D413 carboxylates are important catalytic residues, and their mutations abolish Cas12j3 activity (Fig. 5c–d). Both are predicted to coordinate the metal ions that activate the nucleophile and stabilize the transition state and the leaving group. In our structure, E618 and D413 coordinate the metal and the backbone of the dinucleotide (Fig. 5e, Supplementary Fig. 6). The side chain of D708, which is predicted to act as the third catalytic residue, is not observed due to electron irradiation^36^. This residue has been shown less critical than the other carboxylates in other RuvC domains, as its substitutions to Asn or His lead to a partial loss of cleavage^37,38^. However, in the case of Cas12j3 the D708A mutation abrogates activity (Fig. 5c–d). Structural comparisons using DALI with other RuvC domains support a two-metal ion mechanism (Fig. 6a, Supplementary Table 3).

Taking advantage of the high similarity between Cas12j1, Cas12j2 and Cas12j3 we performed homology modelling using our structure as a template (Methods). The superposition of the models reveals features in the STP domain supporting the different T-strand cleavage kinetics^7^ (Figs. 6c, 1e, Methods). The different insertions in the STP domain observed in Cas12j1 and Cas12j2 may facilitate positioning of the T-strand for cleavage. Although homology is high within the Cas12j family, we did not observe differences between the Cas12j1 and Cas12j2 models with our structure that could explain why Cas12j3 is unable to process its own crRNA, (Fig. 6c, Supplementary Fig. 1).

## Discussion

Genome editing has radically altered life sciences, as the possibility to modify a chosen DNA sequence has triggered a race to find and develop tools useful to conduct a precise and safe modification of a genome. In the past decade, CRISPR-Cas nucleases have been harnessed to target different DNA sequences, triggering a genome editing revolution with stark implications for biomedical and biotechnological applications. However, the large size of these nucleases generates a problem in some of their applications, as the packaging of the current CRISPR-Cas effector complexes into an AAV vector is a limiting factor. In this study, we present the first structural insight of the miniaturized CRISPR-Cas12j family. Due to their reduced size, this group of recently identified compact RNA-guided endonucleases have the potential to alleviate the delivery problems.

Cas12j nucleases display sequence homology only in their RuvC domain with other Class2 types V members^7^. A detailed DALI^39^ search to find structural homologues and compare Cas12j3 with other proteins did not find any homology beyond the RuvC domain (Fig. 6, Supplementary Table 3). In addition, a BLAST search of the whole database for related sequences using the RuvC region (621–647 aa) did not find any homology, suggesting that this feature is only found in members of the Cas12j family. As the Cas enzyme with the shortest protein sequence described to date is Cas12f, we performed a superposition of Cas12j3 with Cas12f using their RuvC domains (Fig. 6a–b, Supplementary Table 3). The Cas12f protein is only 500 residues long; however, the functional RNP consist of a large RNA and two molecules of Cas12f^33^. The Cas12f RNP complex is substantially different to Cas12j3 (Fig. 6b), suggesting that besides the similarities in the catalytic site their working mechanisms should be different.

**Fig. 7:**
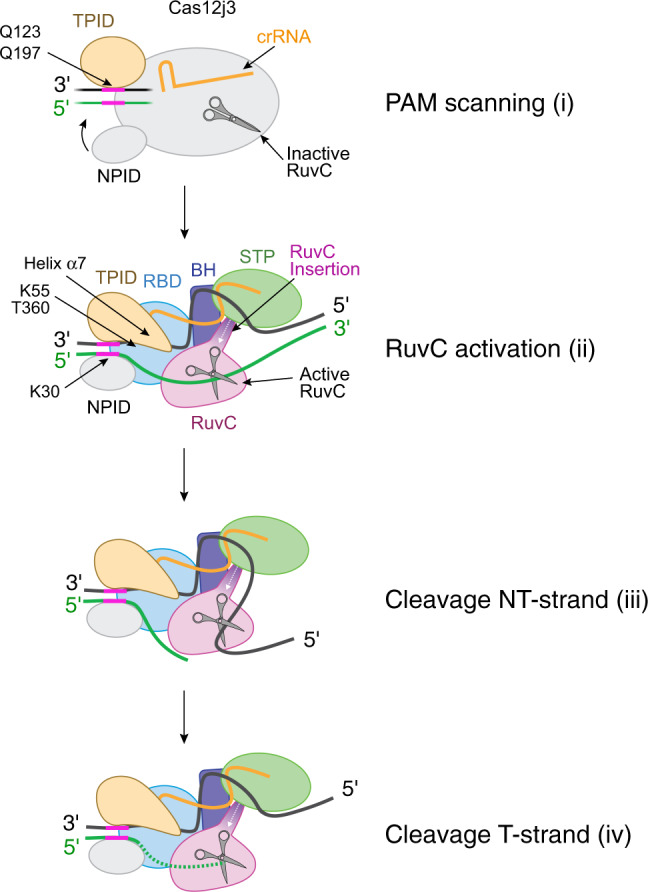
Model of Cas12j3 PAM-dependent DNA recognition, unwinding and cleavage. Cartoon model depicting the stages of Cas12j3 nuclease staggered target DNA cleavage (see Discussion). The T-strand and NT-strand are illustrated in green and black, with the PAM colored in pink. In step (i) the RBD, BH, STP, and RuvC domains are represented as an oval (see Discussion).

In the absence of structural information of the Cas12j3-RNA complex, we can only speculate that the RNP must be in an open conformation in the initial stage of the reaction, in order to allow unwinding of the target DNA. However, the flexibility observed in our cryoEM maps (Fig. 4, Supplementary Movie 1), suggests that after Cas12j3 binds the target DNA (Fig. 7) the PAM scanning would foster the stabilisation of NPID on the NT-strand and the interaction with the TPID would allow PAM binding. Our structure provides us with a snapshot of the R-loop stabilized after cleavage. In this conformation, the hybrid is formed and separated from the NT-strand by the helix α7. We hypothesize that α7 should be in a different conformation to permit dsDNA unwinding and it would clip the hybrid after it has been stabilized by the phosphate inversion. The NT-strand would be directed towards the catalytic site (Fig. 7, Supplementary Movie 1, Supplementary Fig. 7), while the STP domain disrupts the hybrid and orients the T-strand towards the RuvC pocket (Fig. 3 map 2). We propose that the length of the hybrid would be monitored by the RuvC insertion, promoting the activity of the enzyme if the length of the duplex is longer than 12-nt. Catalysis would proceed on the NT-strand, which will access the catalytic pocket with the 5´–3´polarity and later on the T-strand, generating the overhang on the dsDNA. The unspecific ssDNA cleavage activity would be triggered by a similar mechanism after crRNA/target DNA assembly.

Our work provides molecular detail of Cas12j3 PAM recognition, hybrid stabilisation and activation mechanisms. This study paves the way for the rational redesign of these compact RNPs, widening the CRISPR-Cas toolbox. The repurposing of these miniaturized RNA-guided nucleases will open new avenues for their use in genome editing biomedical applications, where the size of the current CRISPR-Cas systems is a challenge.

## Methods

### Plasmid preparation, protein expression and purification

Cas12j3 cDNA was synthetized and cloned with a C-terminal hexahistidine (His)-tag into pET-21 vector (Genewiz) (Supplementary Table 2). Cas12j3 mutants were generated with the In-Fusion cloning kit (Takara) using the primers specified in Supplementary Table II. To generate Cas12j3-∆CT, a TEV cleavage site (ENLYFQG) was generated after the residue M726. His-tagged Cas12j3 was expressed from pET-21 in *E. coli* BL21 pRARE cells. *E. coli* cultures were grown at 37 °C in a liquid Terrific Broth (TB) medium with 34 mg/l chloramphenicol and 100 mg/l ampicillin to an optical density at 600 nm of ~0.8. Both the wild type and mutants were expressed and purified following the same protocol. Only mutations in the insertion turn (W636A, F639A, and F640A) and the STP cleft (W510A, M513A) rendered Cas12j3 insoluble, highlighting the importance of this conserved interaction in the Cas12j family. Overexpression of proteins was induced with 150 nM of IPTG for 16 h at 16 °C. Cells were harvested by centrifugation and resuspended in lysis buffer (50 mM HEPES pH7.5, 2 M NaCl, 5 mM MgCl_2_, 1 tablet of Complete Inhibitor cocktail EDTA Free (Roche) per 50 ml, 50 U/ml Benzonase, 1 mg/ml lysozyme). Lysis was completed by one freeze-thaw cycle and sonication. Cell extract was diluted to a final salt concentration of 500 mM, and high-speed centrifuged (10,000 × g, 45 min) to separate the soluble fraction from the insoluble fraction and the cell debris. The soluble fraction was loaded into a 5 ml HisTrap FF Crude column (Cytiva) equilibrated in buffer IMAC-A (20 mM HEPES pH7.5, 500 mM NaCl, 20 mM Imidazole), and bound proteins were eluted by stepwise increase of the imidazole concentration with buffer IMAC-B (20 mM HEPES pH7.5, 200 mM KCl, 500 mM Imidazole). Cas12j3 proteins eluted at ~ 150 mM Imidazole. In the case of Cas12j3-∆CT, the C-terminal segment (residues 727–766) was cleaved by incubating the protein with 0.3 mg TEV protease in TEV buffer (20 mM HEPES pH 7.5, 150 mM NaCl, 1 mM EDTA, 0.5 mM TCEP) for 16 h at 4 °C. Fractions containing Cas12j3 were pooled, concentrated and further purified by size exclusion chromatography (SEC) using a HiLoad 16/600 Superdex 200 column (Cytiva) equilibrated in SEC buffer (20 mM HEPES pH7.5, 500 mM KCl, 0.5 mM TCEP). Fractions containing pure protein were pooled, concentrated to 5–10 g/L, flash-frozen in liquid nitrogen and stored at −80 °C.

### Cleavage assays

Fluorescein (FAM)-labeled DNA oligonucleotide at 5′ or 3′ ends, unlabeled DNA and RNA oligonucleotides were purchased from Integrated DNA Technologies (IDT) (Supplementary Table II). dsDNA substrates were prepared by mixing ssDNA oligos to a final concentration of 80 µM in annealing buffer (20 mM HEPES pH7.5, 200 mM KCl), denaturation at 95 °C for 10 min and gradually temperature decrease to 4 °C during 20 min in a thermal cycler (Applied Biosystems). Ribonucleoprotein complexes (RNP) of Cas12j3 were formed by mixing an equal volume of 50 µM Cas12j3 and 50 µM Cas12j3 mature crRNA (IDT).

For specific dsDNA cleavage assays, FAM-labeled dsDNA substrates were incubated at 400 nM with 2 µM of Cas12j3 RNP in cleavage buffer (20 mM HEPES pH7.5, 160 mM KCl, 10% glycerol, 5 mM MgCl_2_) for 2 h at 37 °C, or as otherwise stated in the figure legends. For ion dependency assays (Supplementary Fig. 2a) 5 mM MgCl_2_ was substituted by 5 mM Ethylenediaminetetraacetic acid (EDTA), CaCl_2_, MnCl_2_, FeSO_4_, CoCl_2_, NiSO_4_, CuCl_2_, ZnSO_4_. For DNA saturation experiments (Supplementary Fig. 2b) 1 μM of Cas12j3 RNP was incubated with 0.5–8 uM of labelled dsDNA for 2 h at 37 °C. For unspecific trans ssDNA cleavage assays (Fig. 1b–c, Supplementary Fig. 8b–c), 0.4 µM FAM-labeled unspecific ssDNA substrate (i.e., not complementary to the crRNA) was incubated with 2 µM Cas12j3 RNP as described above, along with 0.1 µM of unlabeled activator ssDNA or dsDNA (complementary to the crRNA) in cleavage buffer for 1 h at 37 °C. The reactions were stopped by adding equal volumes of stop buffer (8 M Urea, 100 mM EDTA at pH8) followed by incubation at 95 °C for 5 min. Cleavage products were resolved on 15% Novex TBE-Urea Gels (Invitrogen), run according to the manufacturer’s instructions. Gels were imaged using an Odyssey FC Imaging System (Li-Cor). Densitometric analysis of bands in gels was performed using ImageJ. The cleavage efficiency was calculated as the intensity of the bands corresponding to the products divided by the total intensity for the specific dsDNA cleavage assays, or as the depletion of a signal of the noncleaved product for  unspecific ssDNA degradation assays.

### Sample preparation for Cryo-EM

For the preparation of the Cryo-EM sample, Ni^2+^ was used as a catalytic ion instead of Mg^2+^ due to the higher yield obtained with this metal. Cas12j3 RNP was prepared as described above (cleavage assays). 25 nmol of RNP and 37 nmol of unlabeled dsDNA substrate were incubated in 25 ml of MonoQ A buffer (20 mM HEPES pH7.5, 200 mM KCl, 1 mM NiSO_4_, 0.5 mM TCEP) for 2 h at 20 °C to allow DNA cleavage. The product of the reaction was loaded in a MonoQ column equilibrated with MonoQ A buffer, and Cas12j3/R-loop complex was separated from the RNP and the unbound DNA substrate by a salt gradient elution using MonoQ B buffer (20 mM HEPES pH7.5, 2 M KCl, 1 mM NiSO_4_, 0.5 mM TCEP). Cas12j3/R-loop eluted at 16–20% of MonoQ buffer B (~ 500 mM KCl). The R-loop complex was further purified from unbound DNA by SEC using a Superdex 200 Increase 10/300 GL column (Cytiva) equilibrated with MonoQ A buffer. The molecular weight of the complex and the sample homogeneity was estimated using a Refeyn One mass photometer (Refeyn), using 10–20 nM of protein diluted in MonoQ A buffer (Supplementary Fig. 3a). 2.5 µL of freshly purified Cas12j3/R-loop complex (Absorbance_260 nm_ of ~1.6) was applied to UltrAuFoil 300 mesh R0.6/1.0 holey grids (Quantifoil), glow-discharged for 60 s at 10 mA (Leica EM ACE200), and plunge-frozen in liquid ethane (pre-cooled with liquid nitrogen) using a Vitrobot Mark IV (FEI, Thermo Fisher Scientific) using the next conditions: blotting time 3 s, 100% humidity and 4 °C.

### CryoEM data collection and processing

Movies were collected on Titan Krios G3 Cryo-TEM equipped with a TFS Falcon III camera operated at 300 keV in counting mode. Exposure 1.05 e/Å^2^/frame, in 40 frames and hence a final dose of 42 e/Å^2^. The calibrated pixel size was 0.832 Å/px. All movies were pre-processed using WARP 1.0.9^40^ (Supplementary Fig. 3). Motion correction was performed with a temporal resolution of 20 for the global motion and 5 × 5 spatial resolution for the local motion. We considered motion in the 45–3 Å range weighted with a B-factor of −500 Å^2^. Only Micrographs displaying less than 5 Å intraframe motion were used. CTF estimation was performed using 5 × 5 patches in the 35-4 Å range. We selected micrographs with fitted defocus between 0.0 and 5.0 µm, and a resolution better than 5 Å. For the particle picking, the micrographs were masked, and particles were picked using a re-trained BoxNet deep convolutional neural network. This resulted in 3,504,102 particles from 4393 micrographs. Particles were extracted with a box size of 256 × 256 and a pixel size of 0.832 which were inverted and normalized before being imported into RELION 3.1^41^ for 2D classification. The selected 2D classes were imported in cryoSPARC 3.1.0^42^ where they were 3D classified into four initial classes. The volume with the largest number of particles was refined to an initial 2.61 Å resolution map. The conformational heterogeneity of the particles used in this volume was inspected using the 3D variability analysis in cryoSPARC^24,42^. This method allows the resolution and visualization of the molecular motions taking place in the observed particles. The two more divergent volumes obtained in the 3D variability analysis were used as input for heterogeneous refinement and subsequent non-uniform refinement jobs, yielding maps at 2.6 and 2.8 Å resolution. We further analysed the heterogeneity of the particles used to reconstruct these two maps by performing 3D variability analysis/heterogeneous refinement/non-uniform refinement jobs, resulting in 6 maps at 2.7–3.3 Å resolution (Supplementary Fig. 3d). The three best maps represent the three conformations of the complex that are discussed in the text (depicted as map 1, map 2, and map 3 Supplementary Table 1). Sharpened and local resolution maps were calculated with PHENIX^43^, and directional resolution anisotropy analysis was performed with the 3D-FSC server^44^.

### Atomic model building and refinement

An initial model containing the complete DNA and RNA sequence and ~ 50% of the protein sequence was built ab initio using map-to-model implemented in PHENIX^43^. COOT^45^ was used to connect, extend and correct the protein fragments to generate a model covering ~ 70% of the protein sequence. The rest of the model was auto built by using a buccaneer implemented in CCP-EM^46^ and subsequently corrected in COOT. The final model was obtained after several rounds of refinement using Phenix.real_space_refine and manual inspection and correction in COOT. The final model covers 92% of the protein sequence, mainly lacking a C-terminal segment predicted to be unstructured. Map and molecular model images were created using ChimeraX^47^. The complete polypeptide and R-loop complex were built in the high-resolution map. Besides the flexible N- and C-terminal the only gap in the protein sequence is the loop between residues 160–180. The extra density in the catalytic pocket allowed us to identify the presence of two nucleotides. The density allowed the modelling of dA or dG in the density. We built and a couple of dG nucleotides, in agreement with the abundance of these nucleotides in the NT-strand at that position (Fig. 2b).

### Structural homology models

The high sequence identity between Cas12j1, 2 and 3 (~40%, Supplementary Fig. 1) and the good coverage of the sequence by our structural model allows the generation of structural homology models for Cas12j1 and Cas12j2 with relatively high confidence. These models were generated by using the SWISS-MODEL server (https://swissmodel.expasy.org) using our Cas12j3 structure as template^48^. The results are displayed in (Fig. 6c).

### Superposition of Cas12j3 RuvC domain with other Cas12 enzymes

A DALI^39^ search against the complete PDB did not found homologues of Cas12j3. Only the Cas12j3 RuvC displays homology with the same domain from other Cas12 enzymes (Fig. 6a–b). The Cas12b RuvC domain is the most similar to Cas12j3 (rmsd 4.5 Å for 386 residues), followed by Cas12f and Cas12g (rmsd 4.3 and 4.2 Å for 364 and 327 residues respectively) and Cas12i (rmd 5.1 Å for 383 residues). Cas12 endonucleases generally cleave the T-strand and NT-strand at the single RuvC active site, with the Nuc domain facilitating the loading of the NT- and T-strands into the active site. A BLAST search^49^ of Cas12j3 RuvC insertion (621–647) did not find any similarity in the database, supporting this unique feature of the Cas12j family of endonucleases. Although the region of the protein facilitating the loading of the NT- and T-strand is different to other Cas12 enzymes, the Cas12j family displays 4 strictly conserved Cys residues that bind a metal, which we have modelled as Zn as in the case of Cas12f and Cas12g (Fig. 6a). DALI performed the superposition with Cas12f in the better ordered RuvC, as Cas12f is a dimer. This is the smallest polypeptide found in the CRISPR-Cas family. However, this ~500 residues protein dimerizes upon binding of a large crRNA, which includes a section of the tracr-RNA. Only one of the RuvC domains seems to cleave target DNA^33^.

### Reporting summary

Further information on research design is available in the Nature Research Reporting Summary linked to this article.

## Supplementary information

Supplementary Information

Peer Review File

Description of Additional Supplementary Files

Supplementary Movie 1

Reporting Summary

## Data Availability

The atomic coordinates and cryo-EM maps have been deposited in the Protein Data Bank and EMDB under accession codes 7ODF and EMD-12827. Source data are provided with this paper. All other data are available from the corresponding author upon request. Source data are provided with this paper.

## Source data

Source Data
