# Supplementary material for: Structure of the mini-RNA-guided endonuclease CRISPR-Cas12j3
Source: Nat Commun. 2021 Jul 22;12:4476. doi: 10.1038/s41467-021-24707-3 (PMC8298400; doi:10.1038/s41467-021-24707-3)
Supplement: Supplementary file 1 — Supplementary Information [file 41467_2021_24707_MOESM1_ESM.pdf]

Supplementary. Fig. 1

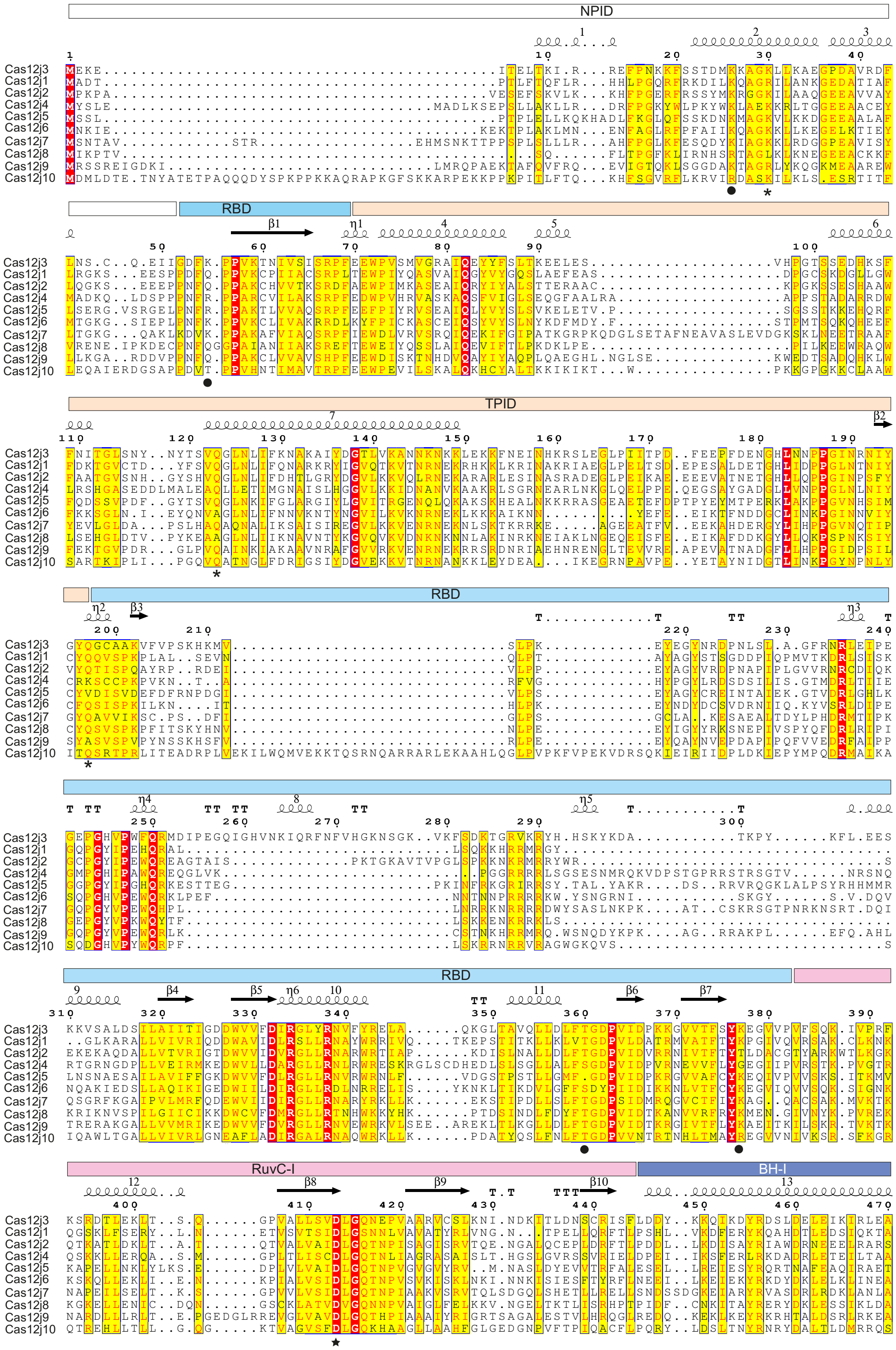

* PAM mutants

● Unwinding mutants

★ Catalytic mutants

■ RuvC insertion mutants

STP and Plug mutants

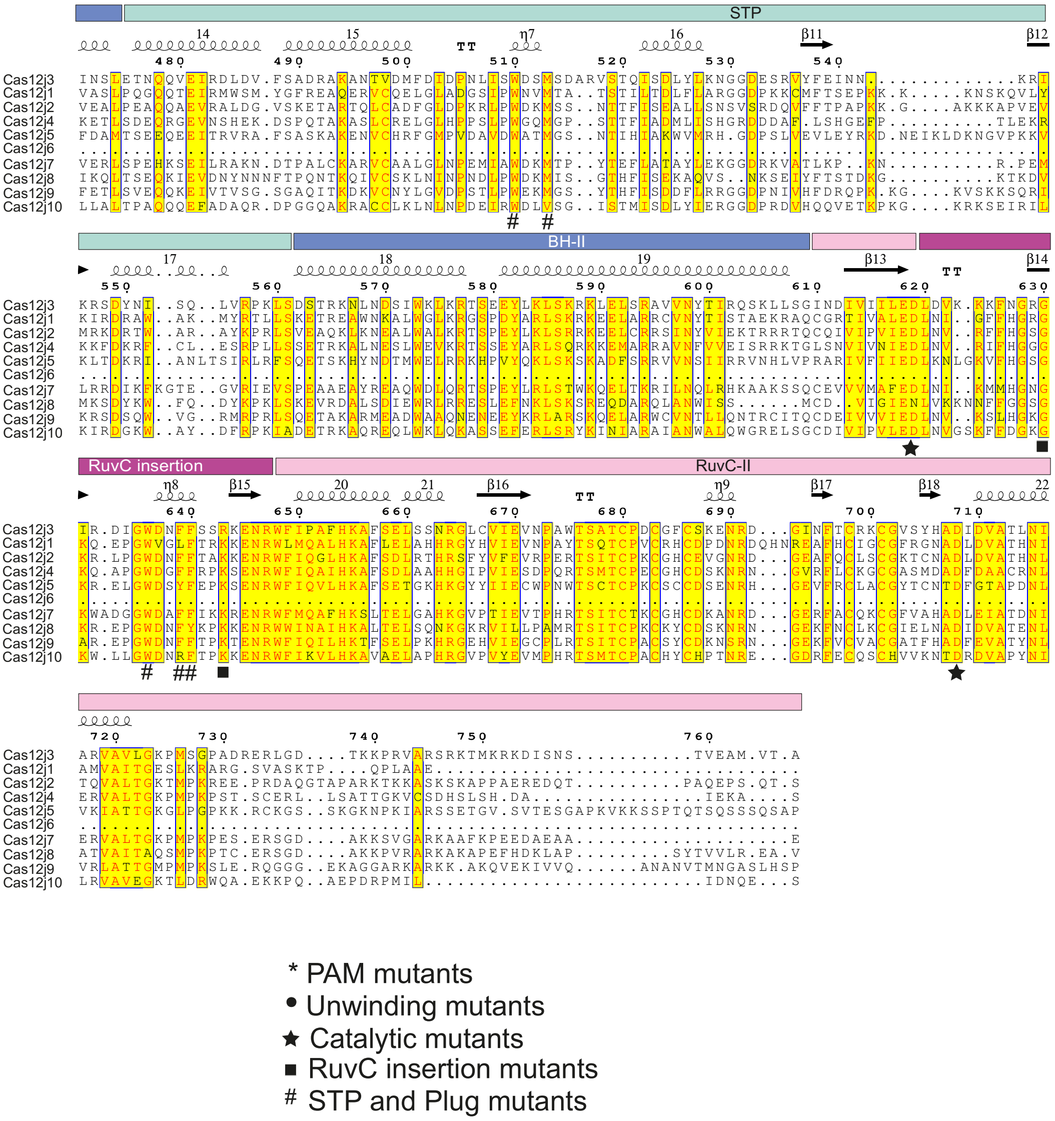

Supplementary Figure 1.- Sequence alignment of known members of the Cas12j family. The amino acid sequences of Cas12j 1 to 10 were aligned by Clustal Omega (http://www.ebi.ac.uk/Tools/msa/clustalo). The figure was prepared with ESPript (http://espript.ibcp.fr). Residue numbers are labelled according to the Cas12j3 sequence. Similar residues are shown in red and identical residues in white over red background. The different structural domains of Cas12j3 and their amino acid composition are depicted as boxes above the sequences and labelled with the same names and colour code as in Fig. 2a. The mutations performed in key residues commented through the text are marked below.

Supplementary Fig. 2

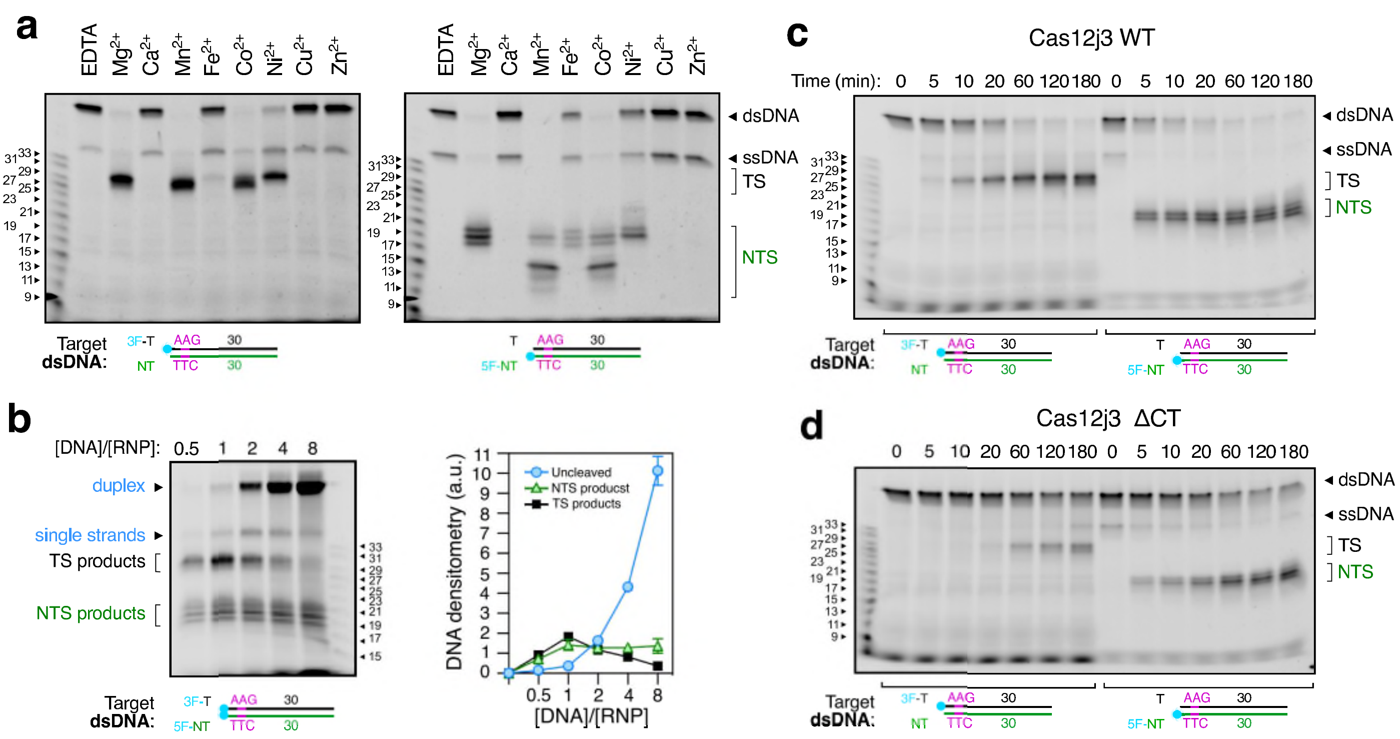

Supplementary Figure 2.- Cas12j3 endonuclease biochemical characterisation. a) DNA cleavage dependency on divalent metal ions. Mg^{2+}, Mn^{2+}, Fe^{2+}, Co^{2+} and Ni^{2+} metal ions support Cas12j3 catalytic activity, while Ca^{2+}, Cu^{2+}, Zn^{2+} do not. Depletion of the cation by EDTA abrogates phosphodiester hydrolysis. **b)** Cleavage assay using the target dsDNA shows the cleavage products of the different strands at different enzyme and substrates ratios. Quantification of the cleaved and non-cleaved dsDNA substrate is shown in the chart as mean ± s.d. (see Methods). The curve shows an increase of the non-cleaved substrate when a 1:1 ratio is reached. An asymptotic behaviour is observed for the NT-strand products. **c)** Time course of the cleavage reaction by Cas12j3. Cas12j3 endonuclease completes the reaction in approximately 120 min for the T-strand while the NT-strand cleavage is completed in 20 min. **d)** Time course of the cleavage reaction by Cas12j3-ΔCT mutant lacking the C-terminal 39 residues. Oligonucleotides 3F-T-AAG-30/NT-TTC-30, T-AAG-30/5F-NT-TTC-30 and 3F-T-AAG-30/5F-NT-TTC-30 (Supplementary Table 2) were used as substrate to visualize the cleavage of the target, non-target and both DNA strands in a-d). Experiments displayed are representative of three replicates. DNA markers are shown in nucleotides.

Supplementary. Fig. 3

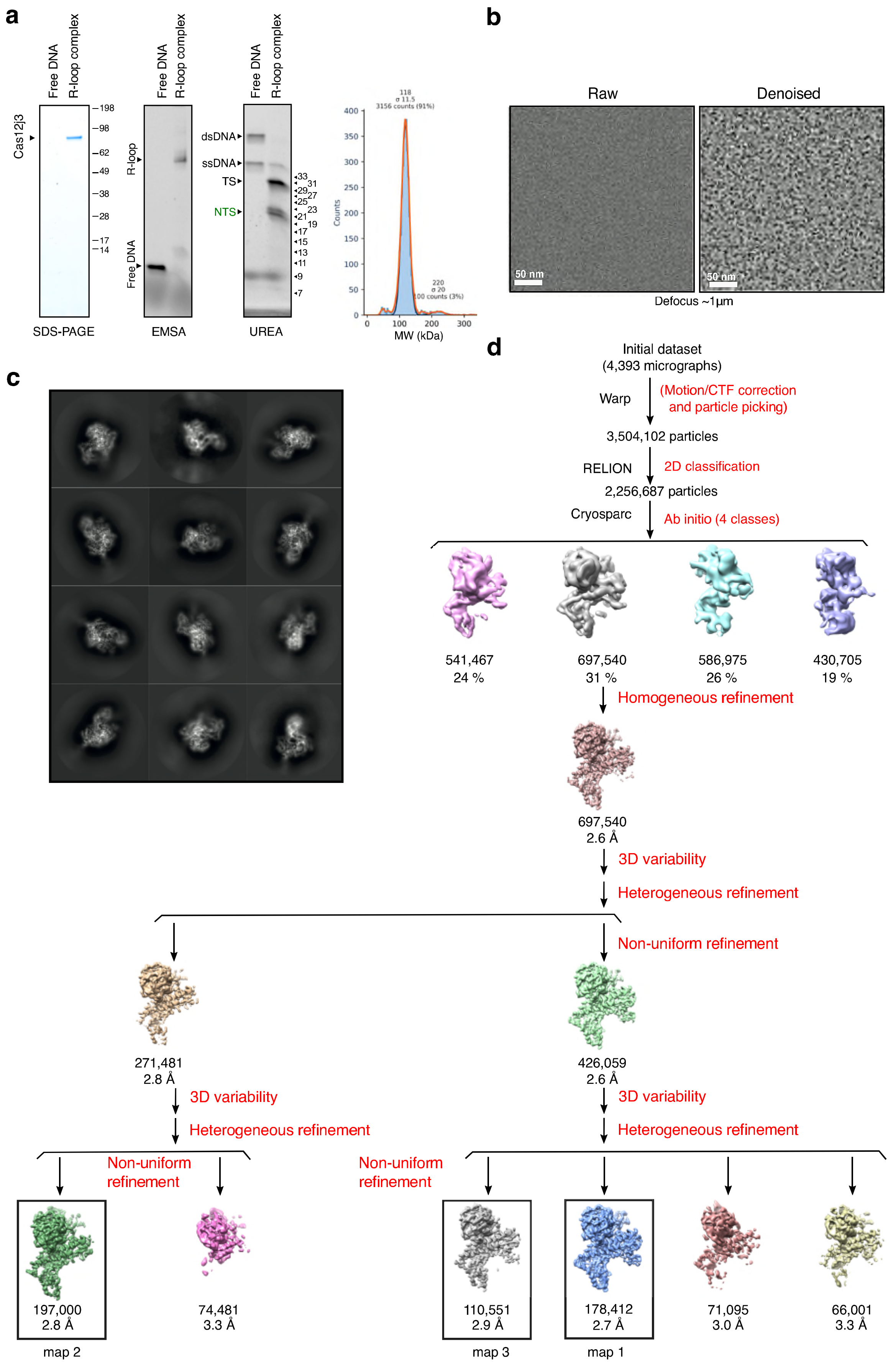

Supplementary Figure 3.- Single particle cryo-EM analysis of the Cas12j3 endonuclease R-loop complex. a) SDS-PAGE, electrophoretic mobility shift assay (EMSA), and denaturing polyacrylamide gels showing the reconstitution of Cas12j3/R-loop complex. The complex shown in a) was reconstituted with the labeled oligonucleotides 3F-T-AAG-30/5F-NT-TTC-30 under similar conditions as the complex used for

cryo-EM. The right-side panel shows the experimental mass and homogeneity of the complex used for cryo-EM (i.e., using the unlabeled nucleotides T-AAG-30/NT-TTC-30), estimated by mass photometry. The position of the protein markers is shown in the SDS-PAGE gel in kDa. The position of the DNA markers is shown in the UREA gel in nucleotides. **b)** Representative cryo-EM micrograph of the Cas12j3/R-loop complex in vitreous ice on gold grids before and after denoising with WARP[1]. Similar particle distribution and ice quality was observed along the 4,393 micrographs used during the processing, and in similar datasets that were not used in the processing. **c)** Representative reference-free 2D class averages sorted by class distribution (RELION [2]). **d)** Overview of the cryo-EM data processing workflow for the Cas12j3/R-loop complex. Similar results of the data processing were obtained with another dataset; however, the ice quality was worse, and it yielded a lower resolution map.

Supplementary. Fig. 4

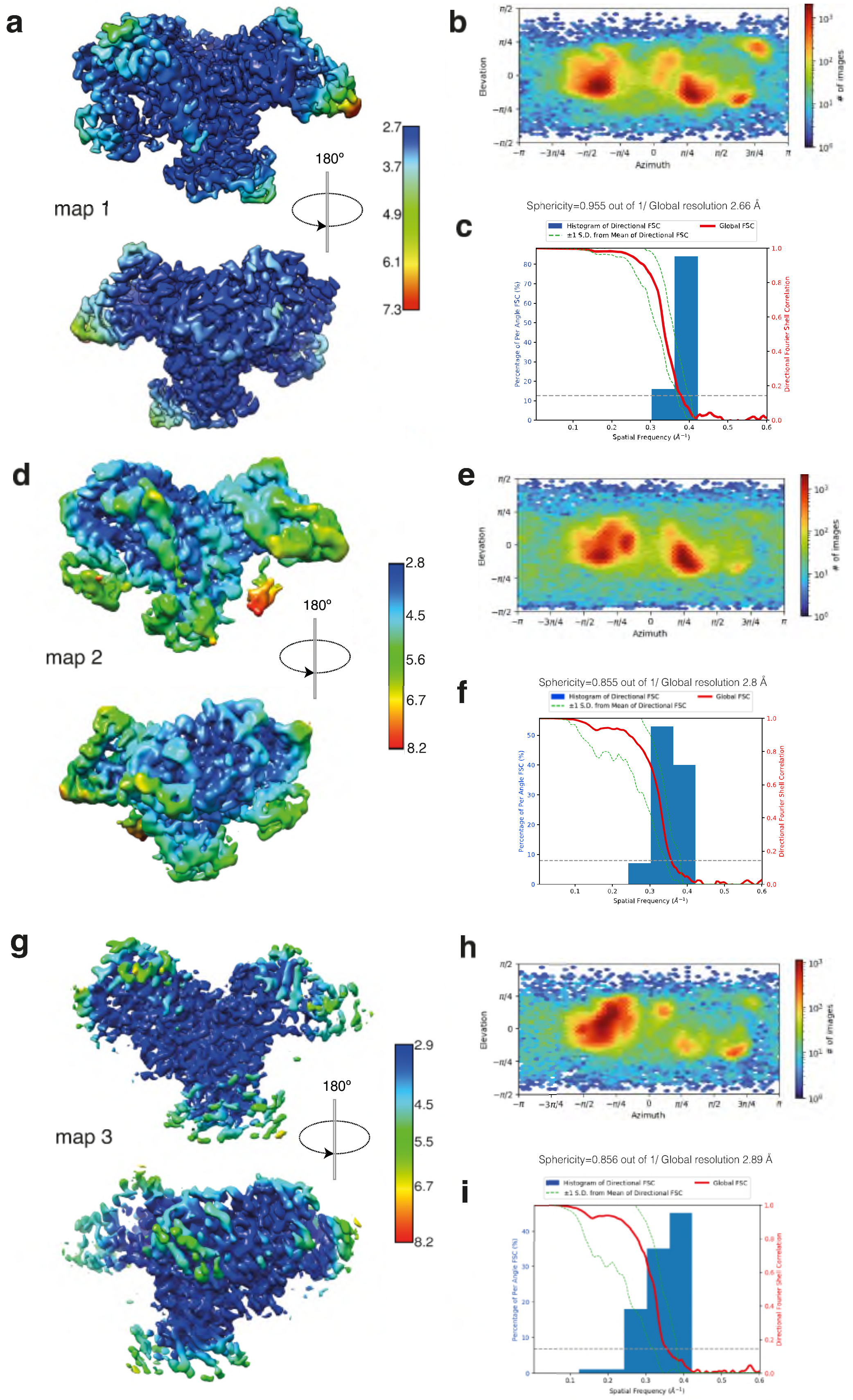

Supplementary Figure 4.- Resolution assessment, validation of cryoEM density maps. a, d, g) Local resolution cryo-EM maps of the Cas12j3-R-loop complex. **b, e, h)** Angular distribution plot showing the

relative orientation of particles in the final 3D reconstruction. **c, f, i)** Fourier shell correlation (FSC) curves and sphericity of the final cryo-EM density maps (3D-FSC[3], Supplementary Table 1).

Supplementary. Fig. 5

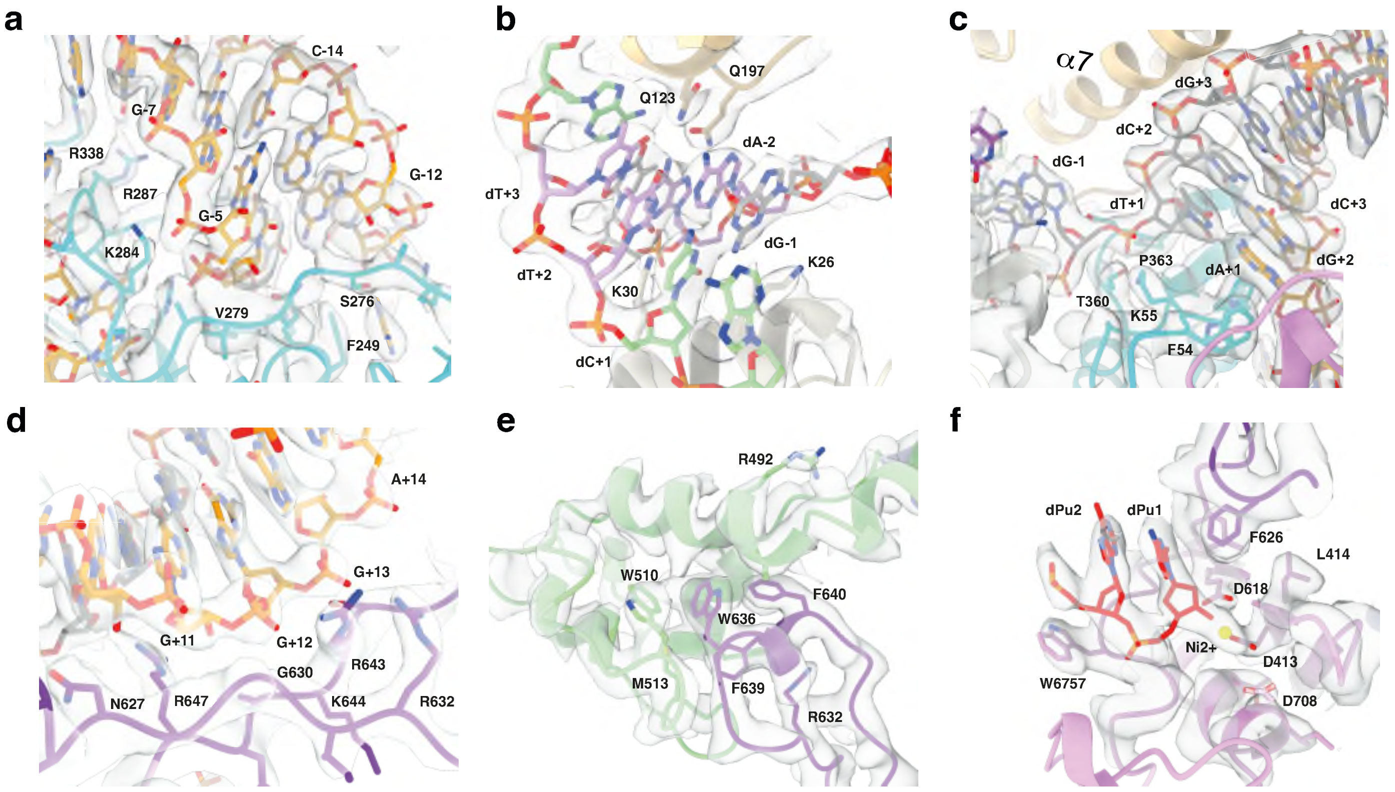

Supplementary Figure 5.- Fit of the model to map 1 in specific regions. a) View of the high-resolution cryo-EM map in the RBD domain (contour level 7.5), **b)** PAM binding region (contour level 7.0), **c)** unzipping cavity (contour level 7.5), **d)** RuvC insertion (contour level 7.8), **e)** hydrophobic plug (contour level 6) and **f)** the RuvC catalytic site (contour level 5.7).

Supplementary. Fig. 6

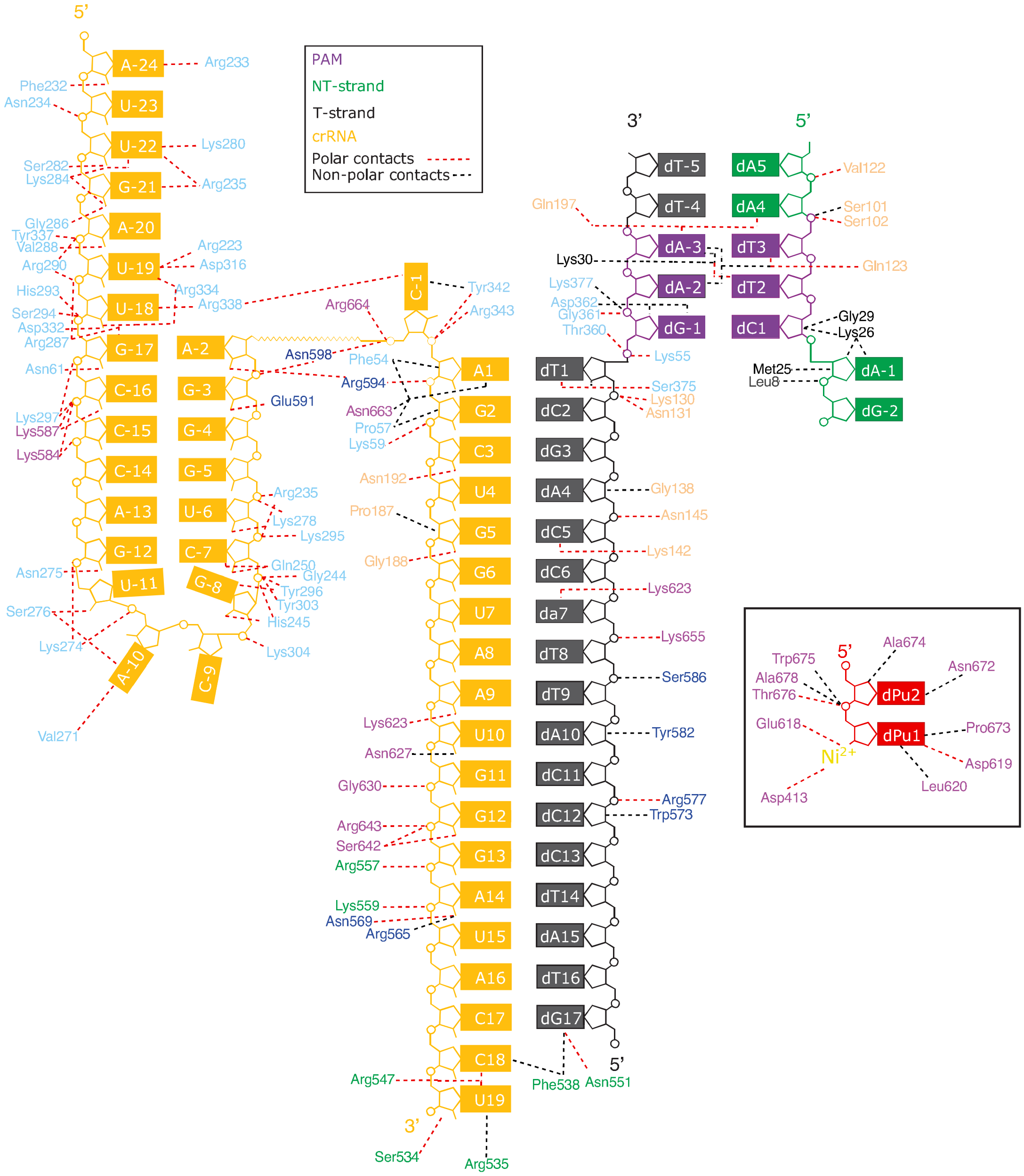

Supplementary Figure 6.- Protein-nucleic acids interactions in the Cas12j3-R-loop structure. Polar and non-polar contacts of the nucleic acids with the protein side and main chain are indicated (see key in the figure).

Supplementary.Fig. 7

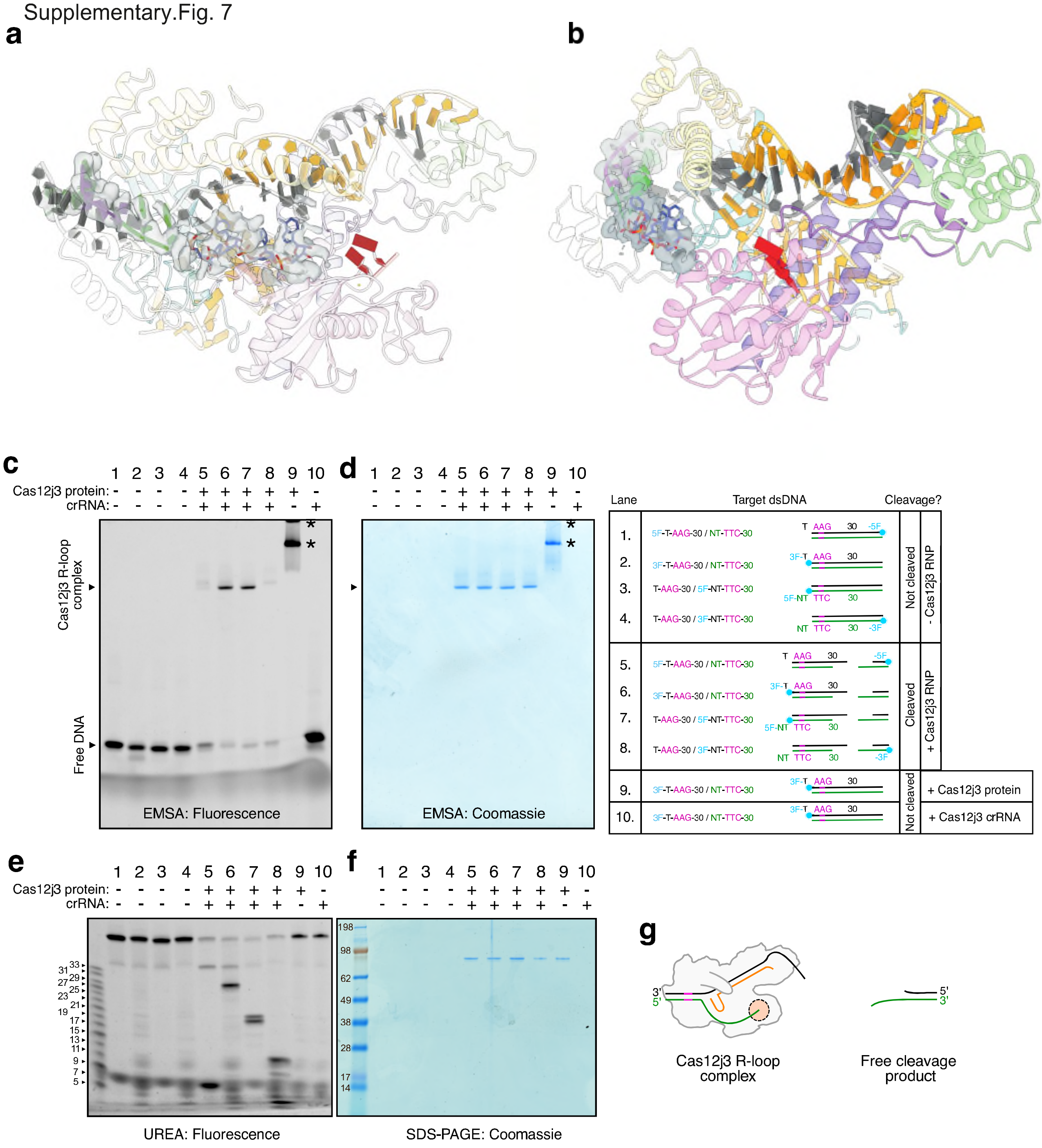

Supplementary Figure 7.- The cleaved NT-strand of the Cas12j3-R-loop complex. a-b) Two different views of the cryo-EM map density observed at low contour suggesting that the dinucleotide in the catalytic

site belongs to the NT-strand. The nucleotides in blue are shown in a) for illustrative purposes only. **c-f)** Binding assay using the NT- and T-strands, FAM labelled in the 5' and 3' positions (for clarity, see table on the right). Lanes 1-4 represent the four free DNAs. Lanes 5-8 show correspond to the R-loop complexes generated after cleavage. Lane 9 corresponds to unspecific complexes (*) formed by the protein and the DNA (no crRNA). Lane 10 is a control experiment including the target dsDNA and the crRNA (no Cas12j3 protein). c) EMSA (electrophoretic mobility shift assay) gel scanned for the FAM fluorescent signal. d) Same gel as shown in c) stained with Coomassie. e) denaturing polyacrylamide gel of the same samples as shown in a) scanned for the FAM fluorescent signal. In e) DNA markers are shown in nucleotides. f) SDS-PAGE of the samples as shown in a) stained with Coomassie. In f) Protein markers are shown in kDa **g)** Cartoon representation of the interpretation of the experiments shown in c-f). After cleavage of the target dsDNA, the PAM-distal fragments are not bound to the complex (lanes 5 and 8), while the PAM-proximal strands remain bound in the R-loop structure. The results shown in c-f) are representative of 3 independent experiments with similar results.

Supplementary Fig. 8.

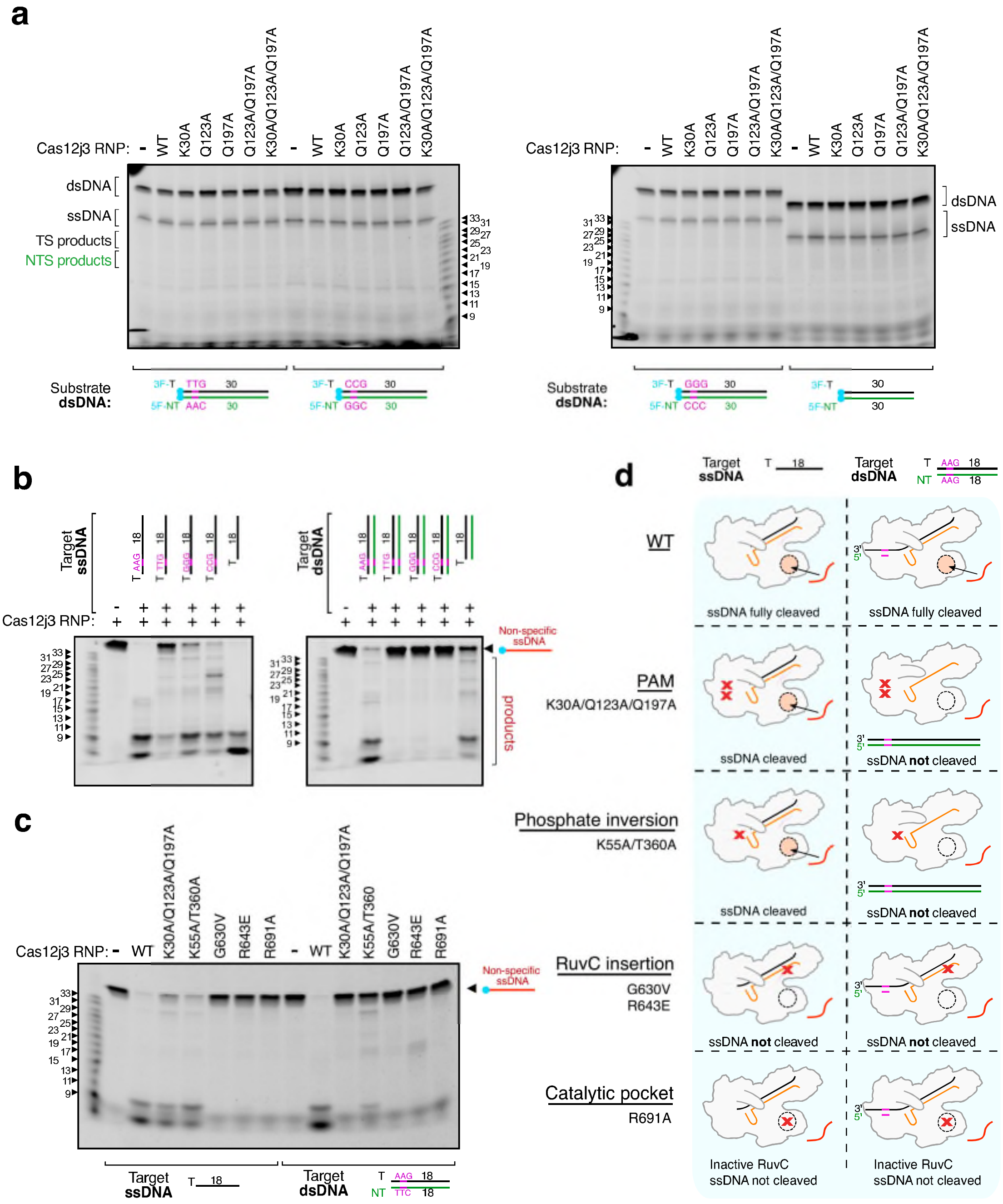

Supplementary Figure 8.- PAM specificity and crRNA/DNA hybrid assembly. a) cleavage assay with Cas12j3 WT and PAM interacting mutants, using target dsDNA as substrate containing different PAM or no PAM sequence. Oligonucleotides 3F-T-AAG-30/5F-NT-TTC-30, 3F-T-TTG-30/5F-NT-AAC-30, 3F-T-CCG-30/5F-NT-GGC-30, 3F-T-GGG-30/5F-NT-CCC-30 and 3F-T-30/5F-NT-30 were used as activators (Supplementary Table II) **b)** Cas12j3 activation of unspecific ssDNA degradation assay using an 18-nt dsDNA

containing different PAM or no PAM sequence as activator. **c)** Unspecific ssDNA degradation by Cas12j3 WT and representative mutants involved in the PAM recognition (K30A/Q123A/Q197A), unwinding (K55A), and RuvC insertion (R643A) after activation with a 18-nt ssDNA without the PAM or a 18-nt dsDNA with the PAM. **d**) schematic representation explaining the results of the experiments shown in c). Gels shown are representative of three independent experiments with similar results. The DNA markers are shown in nucleotides.

Supplementary Tables

Supplementary Table 1. Cryo-EM data collection, refinement and validation statistics

	EMDB-12827 map1 PDB 7ODF	EMDB-12827 map2	EMDB-12827 map3
**Data collection and processing**			
Voltage (kV)	300	300	300
Total electron exposure (e–/Å^2)	42 (1.05/frame)	42 (1.05/frame)	42 (1.05/frame)
Defocus range (μm)	0.8/2.6	0.8/2.6	0.8/2.6
Pixel size (Å)	0.832	0.832	0.832
Symmetry imposed	n/a	n/a	n/a
Initial particle images (no.)	3,504,102	3,504,102	3,504,102
Final particle images (no.)	178,412	197,000	110,551
Sphericity	0.955	0.855	0.856
Map resolution (Å)	2.66	2.80	2.84
FSC threshold	0.143	0.143	0.143
Map resolution range (Å)	2.66-10.19	2.8-11.53	2.87-11.13
**Refinement**			
Initial model used (PDB code)	none		
Model resolution (Å)	2.92		
FSC threshold	0.143		
Model resolution range (Å)	2.7-7.3		
Map sharpening *B* factor (Å^2)	-67.26		
Model composition			
Non-hydrogen atoms	6847		
Protein residues	703		
Nucleotides	78		
Ligands	Ni:1 Zn:1		
Water	5		
B factors (Å^2)	Min/max/mean		
Protein	50.00/144.89/80.86		
Nucleotide	20.00/142.61/79.35		
Ligand	99.25/127.66/113.45		
Water	80.64/80.64/80.64		
R.m.s. deviations			
Bond lengths (Å)	0.004 (0)		
Bond angles (°)	0.827 (0)		
Validation			
MolProbity score	1.35		
Clashscore	6.44		
Rotamer outliers (%)	0.78		
CaBLAM outliers (%)	0.72		
Cβ outliers (%)	0.0		
Peptide plane (%)			
Cis proline/general	0.0/0.0		
Twisted proline/general	0.0/0.0		
Ramachandran plot			
Favored (%)	98.57		
Allowed (%)	1.43		
Disallowed (%)	0.0		

Supplementary Table 2.- Oligonucleotides and primer sequences used in this study

**Oligonucleotides used for Cleavage assays**	
Name	Sequence (5' to 3')
3F-T-AAG-30	GCTGTAGCACAAGGTATCCCATTACCAGCTGAATTAC/36-FAM/
5F-NT-TTC-30	/56-FAM/GTAATTCAGCTGGTAATGGGATACCTTGTGCTACAGC
T-AAG-30-5F	/56-FAM/GCTGTAGCACAAGGTATCCCATTACCAGCTGAATTAC
NT-TTC-30-3F	GTAATTCAGCTGGTAATGGGATACCTTGTGCTACAGC/36-FAM/
T-AAG-30	GCTGTAGCACAAGGTATCCCATTACCAGCTGAATTAC
T-AAG-27	GTAGCACAAGGTATCCCATTACCAGCTGAATTAC
T-AAG-24	GCACAAGGTATCCCATTACCAGCTGAATTAC
T-AAG-21	CAAGGTATCCCATTACCAGCTGAATTAC
T-AAG-18	GGTATCCCATTACCAGCTGAATTAC
T-AAG-15	ATCCCATTACCAGCTGAATTAC
T-AAG-12	CCATTACCAGCTGAATTAC
T-AAG-9	TTACCAGCTGAATTAC
T-AAG-6	CCAGCTGAATTAC
T-AAG-3	GCTGAATTAC
NT-TTC-30	GTAATTCAGCTGGTAATGGGATACCTTGTGCTACAGC
NT-TTC-27	GTAATTCAGCTGGTAATGGGATACCTTGTGCTAC
NT-TTC-24	GTAATTCAGCTGGTAATGGGATACCTTGTGC
NT-TTC-21	GTAATTCAGCTGGTAATGGGATACCTTG
NT-TTC-18	GTAATTCAGCTGGTAATGGGATACC
NT-TTC-15	GTAATTCAGCTGGTAATGGGAT
NT-TTC-12	GTAATTCAGCTGGTAATGG
NT-TTC-9	GTAATTCAGCTGGTAA
NT-TTC-6	GTAATTCAGCTGG
NT-TTC-3	GTAATTCAGC
3F-T-TTG-30	GCTGTAGCACAAGGTATCCCATTACCAGCTGTTTTAC/36-FAM/
5F-NT-AAC-30	/56-FAM/GTAAAACAGCTGGTAATGGGATACCTTGTGCTACAGC
3F-T-CCG-30	GCTGTAGCACAAGGTATCCCATTACCAGCTGCCTTAC/36-FAM/
5F-NT-GGC-30	/56-FAM/GTAAGGCAGCTGGTAATGGGATACCTTGTGCTACAGC
3F-T-GGG-30	GCTGTAGCACAAGGTATCCCATTACCAGCTGGGTTAC/36-FAM/
5F-NT-CCC-30	/56-FAM/GTAACCCAGCTGGTAATGGGATACCTTGTGCTACAGC
3F-T-30	GCTGTAGCACAAGGTATCCCATTACCAGCT/36-FAM/
5F-NT-30	/56-FAM/AGCTGGTAATGGGATACCTTGTGCTACAGC
T-TTG-18	GGTATCCCATTACCAGCTGTTTTAC
NT-AAC-18	GTAAAACAGCTGGTAATGGGATACC
T-GGG-18	GGTATCCCATTACCAGCTGGGTTAC
NT-CCC-18	GTAACCCAGCTGGTAATGGGATACC
T-CCG-18	GGTATCCCATTACCAGCTGCCTTAC
NT-GGC-18	GTAAGGCAGCTGGTAATGGGATACC
T-18	GGTATCCCATTACCAGCT
NT-18	AGCTGGTAATGGGATACC

Non-Specific ssDNA	/56-FAM/ATTTAAATAGGGAAGATAAGCAAAGGGTTGACGAAAGCC
crRNA	**UAUUGAUUGCCCAGUACGCUGGGAC**AGCUGGUAAUGGGAUACCUU
**Oligonucleotides used for cloning and site-directed mutagenesis**	
Name	Sequence (5’ to 3’)
K26A-For	CGATATGGCAAAAGCGGGCAAACTGCTGAAAGCGGAAG
K26A-Rev	CCCGCTTTTGCCATATCGGTGCTGCTAAATTTTTTGTTCGGAAATTCGC
K30A-For	ATGAAAAAAGCGGGCGCACTGCTGAAAGCGGAAGGCCCG
K30A-Rev	TCCGCTTTCAGCAGTGCGCCCGCTTTTTTCATATCGGTG
K55A-For	TTGGCGATTTTGCACCGCCGGTGAAAACCAACATTGTGAGC
K55A-Rev	ccggcggtgcaaaatcgccaaTAATTTCCTGGCAG
Q123A-For	AGCGTGGCGGGCCTGAACCTGATTTTTAAAAACGCG
Q123A-Rev	AGGCCCGCCACGCTGGTATAGTTATAGTTGCTCAGGC
Q197A-For	GGCTATGCGGGCTGCGCGGCGAAAG
Q197A-Rev	GCAGCCCGCATAGCCATAAATGTTGCGGTTAATGCCCG
T360A-For	TGTTTGCCGGCGATCCGGTGATTGATCCGAAAAAAG
T360A-Rev	GCCGGCAAACAGATCCAGCAGCTGCACC
K377A-For	AGCTATGCAGAAGGCGTGGTGCCGGTGTTTAGCCAG
K377A-Rev	CCTTCTGCATAGCTAAAGGTCACCACGCCTTTTTTCGGATCAATCACC
D413A-For	AGCGTGGCCCTGGGCCAGAACGAACCGGTG
D413A-Rev	GCCCAGGGCCACGCTCAGCAGCGCCACC
E618A-For	TTATTCTGGCAGATCTGGATGTGAAAAAAAAATTTAACGGCCGCG
E618A-Rev	CAGATCTGCCAGAATAATCACAATATCGTTAATGCCGCTC
G630A-For	GCCGCGCGATTCGCGATATTGGCTGGGATAACTTTTTTAGCAG
G630A-Rev	GCGAATCGCGCGGCCGTTAAATTTTTTTTTCACATCCAGATCTTCC
R643E-For	GCAGCGAGAAAGAAAACCGCTGGTTTATTCCGGCGTTTC
R643E-Rev	GTTTTCTTTCTCGCTGCTAAAAAAGTTATCCCAGCCAATATCGCGAATGC
R691A-For	AAAACGCCGATGGCATTAACTTTACCTGCCGCAAATGC
R691A-Rev	AATGCCATCGGCGTTTTCTTTGCTGCAAAAGCCGCAATCCG
D708A-For	CTATCATGCGGCTATTGATGTGGCGACCCTGAACATTGCG
D708A-Rev	CATCAATAGCCGCATGATAGCTCACGCCGC
Cas12j3-ΔCT-For	AATCTGTACTTTCAAGGCAGCGGCCCGGCGG
Cas12j3-ΔCT-Rev	TTGAAAGTACAGATTTTCCATCGGTTTGCCCAGCACCG

cDNA sequence of Cas12j3

atggaaaaagaaattaccgaactgaccaaaattcgccgcgaatttccgaacaaaaaatttagcagcaccgatatgaaaaaagcgggcaaactgctgaaagcggaagg
cccggatgcggtgcgcgattttctgaacagctgccaggaaattattggcgattttaaaccgccggtgaaaaccaacattgtgagcattagccgcccgtttgaagaatggc
cggtgagcatggtgggccgcgcgattcaggaatattattttagcctgaccaaagaagaactggaaagcgtgcatccgggcaccagcagcgaagatcataaaagcttttt
taacattaccggcctgagcaactataactataccagcgtgcagggcctgaacctgatttttaaaaacgcgaaagcgatttatgatggcaccctggtgaaagcgaacaaca
aaaacaaaaaactggaaaaaaaatttaacgaaattaaccataaacgcagcctggaaggcctgccgattattaccccggattttgaagaaccgtttgatgaaaacggccat
ctgaacaacccgccgggcattaaccgcaacatttatggctatcagggctgcgcggcgaaagtgtttgtgccgagcaaacataaaatggtgagcctgccgaaagaatat
gaaggctataaccgcgatccgaacctgagcctggcgggctttcgcaaccgcctggaaattccggaaggcgaaccgggccatgtgccgtggtttcagcgcatggatatt

ccggaaggccagattggccatgtgaacaaaattcagcgctttaactttgtgcatggcaaaaacagcggcaaagtgaaatttagcgataaaaccggccgcgtgaaacgct
atcatcatagcaaatataaagatgcgaccaaaccgtataaatttctggaagaaagcaaaaaagtgagcgcgctggatagcattctggcgattattaccattggcgatgatt
gggtggtgtttgatattcgcggcctgtatcgcaacgtgttttatcgcgaactggcgcagaaaggcctgaccgcggtgcagctgctggatctgtttaccggcgatccggtg
attgatccgaaaaaaggcgtggtgacctttagctataaagaaggcgtggtgccggtgtttagccagaaaattgtgccgcgctttaaaagccgcgataccctggaaaaact
gaccagccagggcccggtggcgctgctgagcgtggatctgggccagaacgaaccggtggcggcgcgcgtgtgcagcctgaaaaacattaacgataaaattaccctg
gataacagctgccgcattagctttctggatgattataaaaaacagattaaagattatcgcgatagcctggatgaactggaaattaaaattcgcctggaagcgattaacagcc
tggaaaccaaccagcaggtggaaattcgcgatctggatgtgtttagcgcggatcgcgcgaaagcgaacaccgtggatatgtttgatattgatccgaacctgattagctgg
gatagcatgagcgatgcgcgcgtgagcacccagattagcgatctgtatctgaaaaacggcggcgatgaaagccgcgtgtattttgaaattaacaacaaacgcattaaac
gcagcgattataacattagccagctggtgcgcccgaaactgagcgatagcacccgcaaaaacctgaacgatagcatttggaaactgaaacgcaccagcgaagaatatc
tgaaactgagcaaacgcaaactggaactgagccgcgcggtggtgaactataccattcgccagagcaaactgctgagcggcattaacgatattgtgattattctggaagat
ctggatgtgaaaaaaaaatttaacggccgcggcattcgcgatattggctgggataactttttagcagccgcaaagaaaaccgctggtttattccggcgtttcataaagcgt
ttagcgaactgagcagcaaccgcggcctgtgcgtgattgaagtgaacccggcgtggaccagcgcgacctgcccggattgcggcttttgcagcaaagaaaaccgcgat
ggcattaactttacctgccgcaaatgcggcgtgagctatcatgcggatattgatgtggcgaccctgaacattgcgcgcgtggcggtgctgggcaaaccgatgagcggc
ccggcggatcgcgaacgcctgggcgataccaaaaaaccgcgcgtggcgcgcagccgcaaaaccatgaaacgcaaagatattagcaacagcaccgtggaagcgat
ggtgaccgcgggatccgaattcgagctcgaaaatctgtactttcaaggcgagctccgtcgacaagcttctgctctggaacaccaccatcatcatcactaa

Supplementary Table 3.- DALI search displaying the homology of Cas12j3 with other RuvC family domains.

PDB code	rmsd (Å)	lali	nres	id %	Protein
5u33-A	4.5	386	1085	11	Cas12b
6xmf-A	5.3	384	627	13	Cas12g
7c7l-A	4.3	325	503	17	Cas12f
6w64-A	4.8	376	1037	12	Cas12i
5xuu-A	5.2	347	1213	13	Cas12a
56fa-A	2.6	113	130	16	CNVP61 Holiday Junction resolvase

Supplementary References

1 Tegunov, D. & Cramer, P. Real-time cryo-electron microscopy data preprocessing with Warp. Nature Methods 16, 1146-1152, doi:10.1038/s41592-019-0580-y (2019).

2 Zivanov, J. et al. New tools for automated high-resolution cryo-EM structure determination in RELION-3. Elife 7, doi:10.7554/eLife.42166 (2018).

3 Tan, Y. Z. et al. Addressing preferred specimen orientation in single-particle cryo-EM through tilting. Nat Methods 14, 793-796, doi:10.1038/nmeth.4347 (2017).
