# Supplementary material for: Structure of the mini-RNA-guided endonuclease CRISPR-Cas12j3
Source: Nat Commun. 2021 Jul 22;12:4476. doi: 10.1038/s41467-021-24707-3 (PMC8298400; doi:10.1038/s41467-021-24707-3)
Supplement: Supplementary file 3 — Description of Additional Supplementary Files [file 41467_2021_24707_MOESM3_ESM.pdf]

Description of Additional Supplementary Files

Supplementary Movie 1.- 3D variability analysis showing the conformational heterogeneity of the Cas12j3/R-loop complex. The video displays the movement of the domains in the 3D variability analysis performed with cryoSPARC (Supplementary Fig. 3d, after the volume with 697,540 particles and 2.6 Å resolution). Large variability is observed in the NPID, STP and RuvC domains. The changes observed in the RuvC insertion are concerted with the organisation of the RuvC domain, including the visualisation of the NT-strand and its presence in the catalytic site, as shown in Supplementary Fig. 7. In addition, the STP domain rolls toward the catalytic site pushing the T-strand to the active site. An additional featureless density appears below the STP, which originates from the long T-strand and could be attributed to the long overhang of the T-strand generated after cleavage (Fig. 2b and Fig. 3 map2).
