# Supplementary material for: Structure of the mini-RNA-guided endonuclease CRISPR-Cas12j3
Source: Nat Commun. 2021 Jul 22;12:4476. doi: 10.1038/s41467-021-24707-3 (PMC8298400; doi:10.1038/s41467-021-24707-3)
Supplement: Supplementary file 2 — Peer Review File [file 41467_2021_24707_MOESM2_ESM.pdf]

Editorial Note: This manuscript has been previously reviewed at another journal that is not operating a transparent peer review scheme. The manuscript was considered suitable for publication without further review at *Nature Communications*.
